# Combined analysis of mRNA and miRNA transcriptomes reveals the regulatory mechanism of *Xanthomonas arboricola* pv *pruni* resistance in *Prunus persica*

**DOI:** 10.1186/s12864-024-10113-8

**Published:** 2024-02-27

**Authors:** Pengxiang Zhu, Haiyan Li, Tailiang Lu, Ruizheng Liang, Baoxiong Wan

**Affiliations:** 1 Guangxi Academy of Specialty Crops, Guilin, 541004 China; 2 Guangxi Laboratory of Germplasm Innovation and Utilization of Specialty Commercial Crops in North Guangxi, Guilin, 541004 China

**Keywords:** Peach, *Xanthomonas arboricol*a pv. *pruni*, miRNA, mRNA

## Abstract

**Background:**

Peach bacterial shot hole, caused by *Xanthomonas arboricola* pv *pruni* (Xap), is a global bacterial disease that poses a threat to the yield and quality of cultivated peach trees (*Prunus persica*).

**Results:**

This study compared the mRNA and miRNA profiles of two peach varieties, ‘Yanbao’ (resistant) and ‘Yingzui’ (susceptible), after inoculation with Xap to identify miRNAs and target genes associated with peach tree resistance. mRNA sequencing results revealed that in the S0-vs-S3 comparison group, 1574 genes were upregulated and 3975 genes were downregulated. In the R0-vs-R3 comparison group, 1575 genes were upregulated and 3726 genes were downregulated. Through miRNA sequencing, a total of 112 known miRNAs belonging to 70 miRNA families and 111 new miRNAs were identified. Notably, some miRNAs were exclusively expressed in either resistant or susceptible varieties. Additionally, 59 miRNAs were downregulated and 69 miRNAs were upregulated in the R0-vs-R3 comparison group, while 46 miRNAs were downregulated and 52 miRNAs were upregulated in the S0-vs-S3 comparison group. Joint analysis of mRNA and miRNA identified 79 relationship pairs in the S0-vs-S3 comparison group, consisting of 48 miRNAs and 51 target genes. In the R0-vs-R3 comparison group, there were 58 relationship pairs, comprising 28 miRNAs and 20 target genes. Several target genes related to resistance, such as *SPL6*, *TIFY6B*, and *Prupe.4G041800_v2.0.a1* (PPO), were identified through literature reports and GO/KEGG enrichment analysis.

**Conclusion:**

In conclusion, this study discovered several candidate genes involved in peach tree resistance by analyzing differential expression of mRNA and miRNA. These findings provide valuable insights into the mechanisms underlying resistance to Xap in peach trees.

**Supplementary Information:**

The online version contains supplementary material available at 10.1186/s12864-024-10113-8.

## Background

 The pathogen of bacterial shot hole disease in peach trees is *Xanthomonas arboricola* pv. *pruni* (Xap). This disease has the potential to cause severe economic losses, as it can easily spread and affect a large number of peach trees [1]. Therefore, it is of great value and significance to clarify the mechanism of peach germplasm resources resistant to Xap, which will lay a foundation for the genetic improvement of peach to achieve both high yield and disease resistance.

In recent years, continuous development of high-throughput sequencing technology has made it a mature technology to analyze the expression of plant genes under different conditions and identify genes that confer disease resistance. In sugar beet, 50 differentially expressed genes related to infection defense were successfully identified by comparing normal and infected bacterial gene expression profiles [2]. In Arabidopsis, gene knockout technology revealed that *RBOH1* is a gene involved in Arabidopsis resistance to *Botrytis cinerea* [3]. In grapes, the MYB transcription factor family plays a significant role in grape resistance to downy mildew, as found through RNA-Seq technology [4]. However, there are limited studies on the transcriptome concerning stress resistance during peach development. Sun et al. compared the responses of two peach varieties GF677 and Mao peach to saline-alkali stress and identified several genes and pathways related to the differential tolerance of this stress [5]. Niu et al. investigated the molecular response of peach trees to the feeding of peach aphids and found that the resistance gene Rm3 activated defense-related pathways and signal elements, leading to the production of specific secondary metabolites that affect the interaction of peach aphids [6]. Rubio et al. have shown that by activating ribonucleic acid silencing mechanism and regulating the expression of defense response, disease-related proteins and plant hormone signal transduction related genes, “Garigos” apricot scion grafted on “GF305” peach rootstock can induce peach resistance to *plum pox virus* (PPV) [7]. Svetaz et al. discussed the pathogenicity and plant response mechanism of resistant and susceptible peach varieties to amoeba fungi, and revealed the commonness and difference of peach trees in the process of infection and early events [8].Recent studies have shown that some genes are involved in resistance to bacterial wilt [9]. The transcriptome of *Monilinia fructicola* during different stages of peach brown rot infection has been studied in several research articles [10, 11]. Research related to plant disease resistance provides a detailed picture of how gene expression is regulated under specific pathogen infections, which helps to deepen our understanding of the molecular mechanisms underlying the interactions between plants and pathogens [12–15]. These studies explore information related to signal pathways, transcription factor networks, metabolic pathways, and protein interactions associated with plant defense responses, thereby revealing how plants respond to attacks from different types of pathogens by changing gene expression [16–19]. For peach bacterial shot holes, it is particularly important to study the transcriptome to understand how cells respond to Xap infection and defense mechanisms to further develop methods to control the disease.

Transcriptome and miRNA research are often intertwined. While delving into transcriptome analysis, the role of miRNA in regulating protein-coding genes should also be considered. Similarly, while exploring miRNA function, the impact of the transcriptome needs to be taken into account to better explain the mechanism of action of miRNAs. miRNAs are a class of small RNAs that are ubiquitous in various organisms. Studies have shown that miRNAs play important biological roles in many processes, including gene regulation, cell growth and differentiation, and the immune response. Recent studies have also indicated that many plant miRNAs are associated with plant disease resistance [20]. These miRNAs can affect the function of the plant’s immune system by regulating the expression of target genes, thereby improving plant resistance to pathogens [21–23]. miRNAs play an important regulatory role in the interaction between plants and viruses. When plant viruses infect, they invade RNA into host plant cells and use the mechanism of plant cells to replicate and transcribe [24]. During fungal infections, miRNAs can bind to fungal mRNA, mediate its degradation or inhibit translation, thereby regulating fungal gene expression [25]. miRNAs may also directly target plant transcription factors such as WRKY and MYB transcription factors, which are essential for the plant immune response [26, 27]. miRNAs can also affect the expression of antifungal-related proteins in plants [28].

miRNAs can regulate gene expression and control various physiological processes during plant growth and development, including root differentiation, nutrient absorption, flowering, and fruit development [29–33]. Additionally, miR398 regulates the antioxidant capacity of plants by targeting the expression of copper-binding proteins under oxidative stress conditions [34]. In addition, miRNAs participate in the metabolic regulation of plants by affecting the production of flower color and aromatic substances through the regulation of the flavonoid metabolic pathway [35]. Small RNA sequencing (sRNA-seq) of samples from the leaves of SMD-susceptible variety ROC22 and SMD-resistant variety FN39 infected by SrMV was performed [36]. Lü et al. summarized the regulatory roles of miRNAs in plant‒pathogen and plant-probiotic interactions [37]. Using the available knowledge in rice and other model plants, the important roles of miRNAs in regulating host responses to various fungal, bacterial, and viral pathogens and insect pests in the context of gains and trade-offs to crop yield have been examined [38]. Yan et al. studied WRKY genes to provide novel insights into their role against *Ralstonia solanacearum* infection in cultivated peanut (*Arachis hypogaea* l.) [39]. A total of 174 WRKY genes (*AhWRKY*) were identified from the genome of cultivated peanuts. Liao et al. aimed to provide a comprehensive view describing the new molecular mechanism associated with miR482/2118 in the plant immune system [40]. The study of miRNAs has made remarkable progress, and miRNAs are also involved in the regulation of disease resistance in many plants [41–48]. Research on fruit tree miRNAs started late, but with the increasing maturity of next-generation sequencing technology for analyzing small RNA sequences, numerous miRNA sequences have been obtained for fruit trees such as grapes [49], citrus [50], apples [51], peaches [52], and papayas [53]. In conclusion, research on miRNAs in plants has made great progress, and there is still much work to be done in the future to better understand the regulatory mechanism of miRNAs in plants and their relationship with growth and development.

At present, there is a lack of reports on the response of peach to Xap infection. The key objective of this study is to determine the miRNAs and target genes associated with peach tree resistance by comparing the mRNA and miRNA expression profiles of resistant and susceptible peach tree varieties after inoculation with Xap. The innovation of this study lies in the combination of mRNA and miRNA differential expression analysis, which identifies several candidate genes that may be involved in peach tree resistance. Transcriptome and miRNA combined analysis was used to reveal the main response pathways under Xap infection. The findings provide valuable theoretical references for the genetic improvement of peach disease resistance. It is essential to identify and screen peach resistance resources in China and study the molecular mechanism of peach bacterial perforation resistance. Exploring key resistance-related genes is of great significance because it provides theoretical support for the final resistance breeding process.

## Materials and methods

### Plant material and growth conditions

One-year-old seedlings of *P. persica* were kindly provided by a peach garden (located in Guilin city, Guangxi Zhuang Autonomous Region, China, 110°59′N, 25°39′E).

A peach cultivar ‘Yanbao’ (R) resistant to *X.arboricola* pv *pruni* (Xap) was selected and bred by the Hebei Academy of Agricultural and Forestry. The approval number was “Ji S-SV-PP-015-2011”, and the other susceptible variety was ‘Yingzui’ (S). This is a traditional local variety that was collected and preserved by the Guangxi Academy of Specialty Crop. Both resources are also held at the National Horticulture Germplasm Resources Centre and are available for sharing and free of charge. In this study, we selected 3 healthy 1-year-old peach trees from each variety of peaches grown in open fields. From each tree, we selected 3 young and tender leaves from different branches. The leaves were then inoculated with Xap using the needle puncture method. After activation, the Xap was cultured in LB liquid medium at 28 °C for 48 h. The liquid medium was then discarded after centrifugation at 4000 rpm. The bacterial cells were suspended in sterile water and set aside.

For each sample, including ‘Yanbao’ (uninoculated with Xap) at 0 days (R0), Xap inoculated at 3 days (R3), and 5 days (R5); and ‘Yingzui’ (uninoculated with Xap) at 0 days (S0), Xap inoculated at 3 days (S3), and 5 days (S5), we collected a total of 6 groups of samples. Each sample was a mixture of 5 independent plants and the experiment was repeated 3 times. Consequently, we obtained a total of 18 samples, which were stored at -80 °C.

### Transcriptome sequencing

Total RNA was extracted using a TaKaRa MiniBEST Plant RNA Extraction Kit (TaKaRa, Dalian,China) according to the manufacturer’s protocol. RNA quality was assessed using a Nanodrop™ 2000 spectrophotometer (Thermo Fisher Scientific, USA) and checked by 1% agarose gel electrophoresis. After extraction of total RNA, eukaryotic mRNA was enriched using oligo (dT) beads. The enriched mRNAs were fragmented into short RNAs using fragmentation buffer and reverse transcribed into cDNA by using the NEBNext Ultra RNA Library Prep Kit for Illumina (NEB #7530, New England Biolabs, Ipswich, MA, USA). The double-stranded cDNA fragments were purified, end repaired, a base added, and ligated to Illumina sequencing adapters. The ligation reaction was purified with AMPure XP Beads (1.0X). The ligated fragments were subjected to size selection by agarose gel electrophoresis and polymerase chain reaction (PCR) amplification [17]. The resulting cDNA library was sequenced using Illumina NovaSeq 6000 by Gene Denovo Biotechnology Co. (Guangzhou, China).

### Sequencing data quality control and alignment analysis

In order to ensure the quality of the data for this study, the raw data is filtered prior to analysis to reduce the interference caused by invalid data. Firstly, we use fastp to perform quality control on the raw reads obtained from the sequencing process, resulting in clean reads. The steps for filtering the reads are as follows: Remove reads containing adapters; exclude reads with an N ratio greater than 10%; remove reads consisting entirely of “A” bases; eliminate low-quality reads, where the number of bases with a quality value (Q) of ≤ 20 accounts for more than 50% of the entire read.

In this study, the reference genome used is “Prunus_persica_genome_v2.0.a1”. We performed alignment analysis based on the reference genome using the HISAT2 software.

### microRNA Library construction and sequencing

After total RNA was extracted by a TRIzol reagent kit (Invitrogen, Carlsbad, CA, USA), RNA molecules in a size range of 18–30 nucleotides (nt) were enriched by polyacrylamide gel electrophoresis (PAGE). The 36–48 nt RNAs were also enriched. The 3’ adapters were added to all the enriched RNAs. The 5’ adapters were then ligated to the RNAs as well [42]. The ligation products were reverse transcribed by PCR amplification, and the 140–160 bp PCR products were enriched to generate a cDNA library, which was sequenced using Illumina NovaSeq 6000 by Gene Denovo Biotechnology Co. (Guangzhou, China).

### The bioinformatics analysis of miRNA sequences

To ensure the accuracy of the subsequent assembly and analysis, the raw reads obtained from the sequencing machines were filtered. This involved removing reads with low quality bases or adapters, as well as those without miRNA fragments between adapters. Additionally, reads containing polyA and those shorter than 18nt were also eliminated. The resulting clean tags were then compared against the miRBase database (Release 22) to identify known miRNAs for the species being studied. Any clean tags that did not have a match in the database were aligned with the reference genome. By considering their genome positions and hairpin structures predicted by software miRDeep2, novel miRNA candidates were identified.

### Identification of differentially expressed mRNAs and miRNAs

To identify differentially expressed transcripts across samples or groups, the DESeq2 software was used. EdgeR software was used to identify miRNA differential expression analysis between samples or between groups. Significant differential expression of mRNAs and miRNAs was determined based on fold change and statistical significance. FDR < 0.05(mRNA), |log2FC|>log2.*p* < 0.05.

### Prediction of miRNA targets

The software patmatch (http://www.arabidopsis.org/cgi-bin/patmatch/nph- patmatch.pl) was used to predict miRNA target genes. miRNA sequences and family information were obtained from the TargetScan website (http://www.targetscan.org/).

### Construction of the miRNA‒target network

The expression correlation between miRNA targets was evaluated by means of Pearson correlation coefficient (PCC). For multiple groups, pairs with PCC < -0.7 and *p* < 0.05 were selected as negatively coexpressed miRNA‒target pairs.

### Visualization of the miRNA‒target network

The miRNA‒target network was constructed as above and then visualized using Cytoscape software (v3.6.0) (http://www.cytoscape.org/).

### Functional enrichment analysis

Gene Ontology (GO) Biological Processes term and Kyoto Encyclopedia of Genes and Genomes (KEGG) pathway analyses were performed to assess functional enrichment [54].

### qRT-PCR validation

According to the HiScript® Q Select RT SuperMix for qPCR (+ gDNA wiper) Reverse Transcription Kit (Nanjing Vazyme Biotech Co., Ltd. China), total RNA was reverse transcribed into cDNA using the Bio-Rad CFX96 instrument. The cDNA (2 µL), forward primer (0.4 µL, 10 µM), reverse primer (0.4 µL, 10 µM), 2× ChamQ™ SYBR® qPCR Master Mix (10 µL), and RNase-free ddH_2_O (7.2 µL) were combined to a total volume of 20 µL for further experimentation. The amplification program was as follows: initial denaturation at 95℃ for 30 s, followed by 40 cycles of denaturation at 95℃ for 10 s and annealing/extension at 60℃ for 30 s. A final extension step was performed at 95℃ for 15 s, followed by a melt curve analysis at 60℃ for 60 s and a final hold at 95℃ for 15 s. Each experiment included three biological replicates. PREDICTED: Prunus persica actin-7 and 5.8 S rRNA were used as reference genes.

### Statistical analyses

TBtools software was used to generate the heat map [55]. One-way ANOVA followed by Dunnett’s multiple comparison test was performed using GraphPad Prism version 8.0.0 for Windows (www.graphpad.com; GraphPad Software, San Diego, California USA). Table S1 lists all the primers used in the qRT‒PCR experiments.

## Results

### Comparison of disease severity on peach leaves with different resistance levels after Xap inoculation

**Fig. 1:**
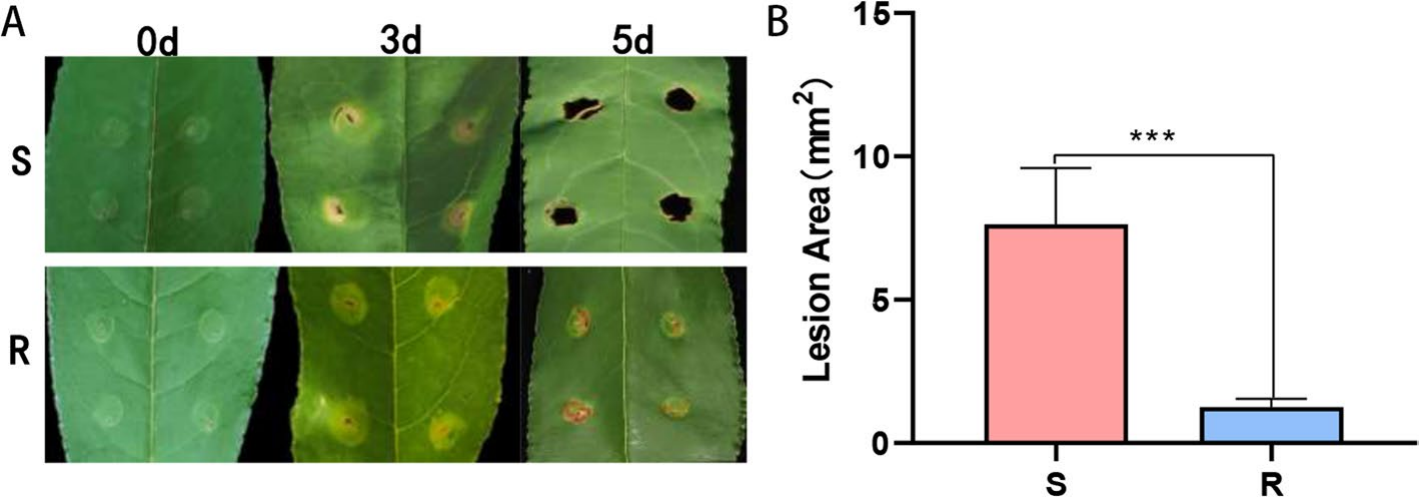
**A** Symptoms of peach leaves after 3d and 5d of inoculation. **B** Statistics of lesion area of different varieties. The error line represents the standard deviation of three biological replicates. The t-test was used to test the significance of the difference in data, and * * * indicated *p* < 0.001

To evaluate the resistance of the peach variety ‘Yanbao’ to Xap, this study selected the peach variety ‘Yingzui’ with a lower resistance level to Xap as the control. After inoculation of Xap on the leaves of two peach varieties, it was found that susceptible ‘Yingzui’ had early onset, rapid development, and a severe degree of Xap-associated symptoms, while resistant ‘Yanbao’ had late onset, slow development and a mild degree of Xap-associated symptoms. As shown in Fig. 1, obvious symptoms were observed on both peach leaves at 3 days postinoculation (dpi). Necrotic lesions began to appear and spread rapidly over time, and the lesion area on the leaves of ‘Yingzui’ was 61% larger than that of ‘Yanbao’ at 5 dpi.

### Transcriptome sequencing data statistics of peach leaves after inoculation

Peach leaves were selected at 0 d, 3 d, and 5 d after Xap inoculation as the materials for transcriptome sequencing. Illumina NovaSeq 6000 platform sequencing generated a total of 820,129,486 original reads from the six cDNA libraries. After removing low-quality reads, a total of 816,943,982 net reads were obtained. Q20 values were over 97% for all six samples, indicating high sequencing accuracy. The percentage of N content, representing the proportion of unknown bases in the sequence, was as low as 0.00%. Additionally, the percentage of GC content ranged from 45.56 to 46.07% (Table S2).

Comparison results between the sequencing data and the selected reference genome data are presented in Table S3. The comparison efficiency between the sequenced data of each sample and the reference genome sequence was over 90%, revealing that the difference between the materials used in this study and the reference genome is small. Such comparability can meet the requirements for subsequent data analysis.

The results of Principal Component Analysis (PCA) are shown in Fig. S1A, where the first two principal components (PC1, PC2) effectively distinguish different varieties and treatments. Additionally, the sample correlation results are presented in Fig. S1B, indicating a high correlation between biological replicates within the same group, implying good reproducibility within the sample groups.

### Statistical analysis of differentially expressed genes in resistant and susceptible varieties

**Fig. 2:**
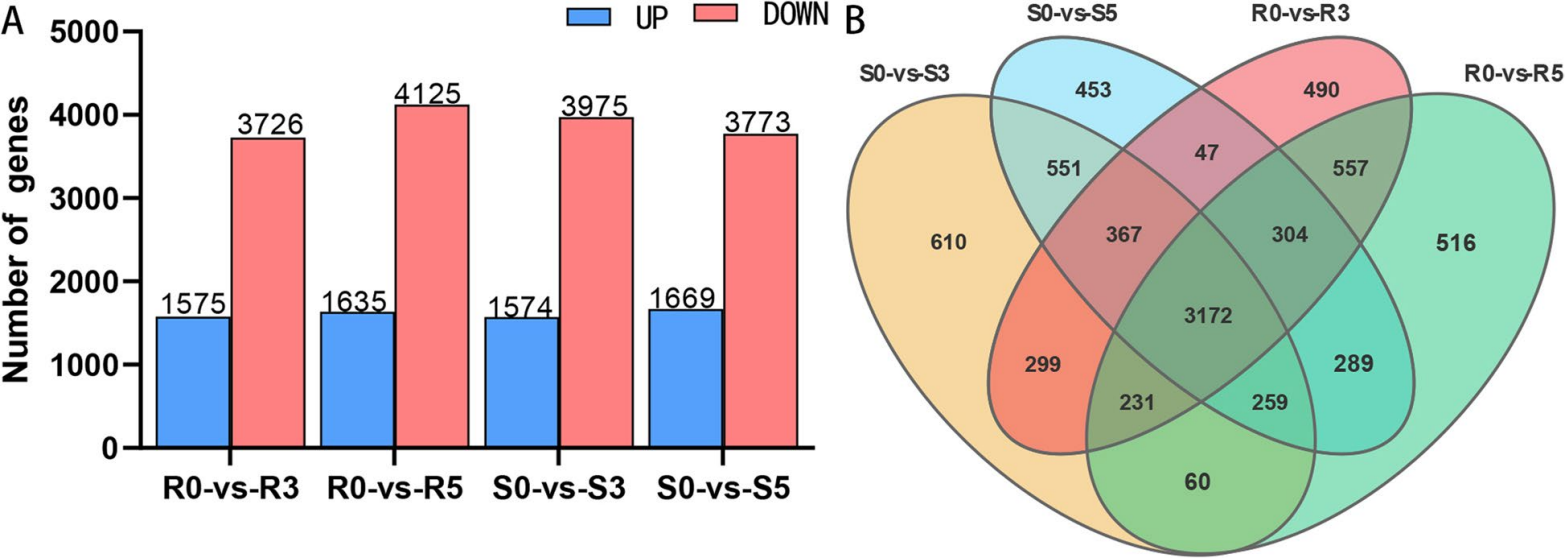
**A** The number of differential genes in the comparison group. **B** Wayne diagram of differentially expressed genes in the comparison group

DESeq2 software was utilized for analysis to screen significantly differentially expressed genes. The results are shown in Fig. 2A. In the Venn diagram (Fig. 2B) of three treatment groups for disease-resistant varieties compared to the control group, it was found that there were 3172 common differentially expressed genes among the four comparison groups (R0vsR3, R0vsR5, S0vsS3, and S0vsS5). These common DEGs are unrelated to the infection time point, representing the difference between resistant and susceptible varieties, and are not related to Xap infection. A volcano map of differentially expressed genes was also generated, depicting differences in gene expression levels and statistical significance of the differences between the two samples and the control group (Fig. S2A-D).

Overall, as infection time increased in susceptible varieties, the number of differentially expressed genes increased, with the largest increase observed on day 5, but the number of downregulated genes gradually decreased. In contrast, both upregulated and downregulated genes in disease-resistant varieties gradually increased over time. When comparing gene expression between resistant and susceptible varieties at the same sampling time point, the number of differentially expressed genes initially increased and then gradually decreased, going from 1368 at 0 d to 1451 at 3 d and then dropping to 1202 at 5 d.

### Significant enrichment analysis of GO function of differential genes

**Fig. 3:**
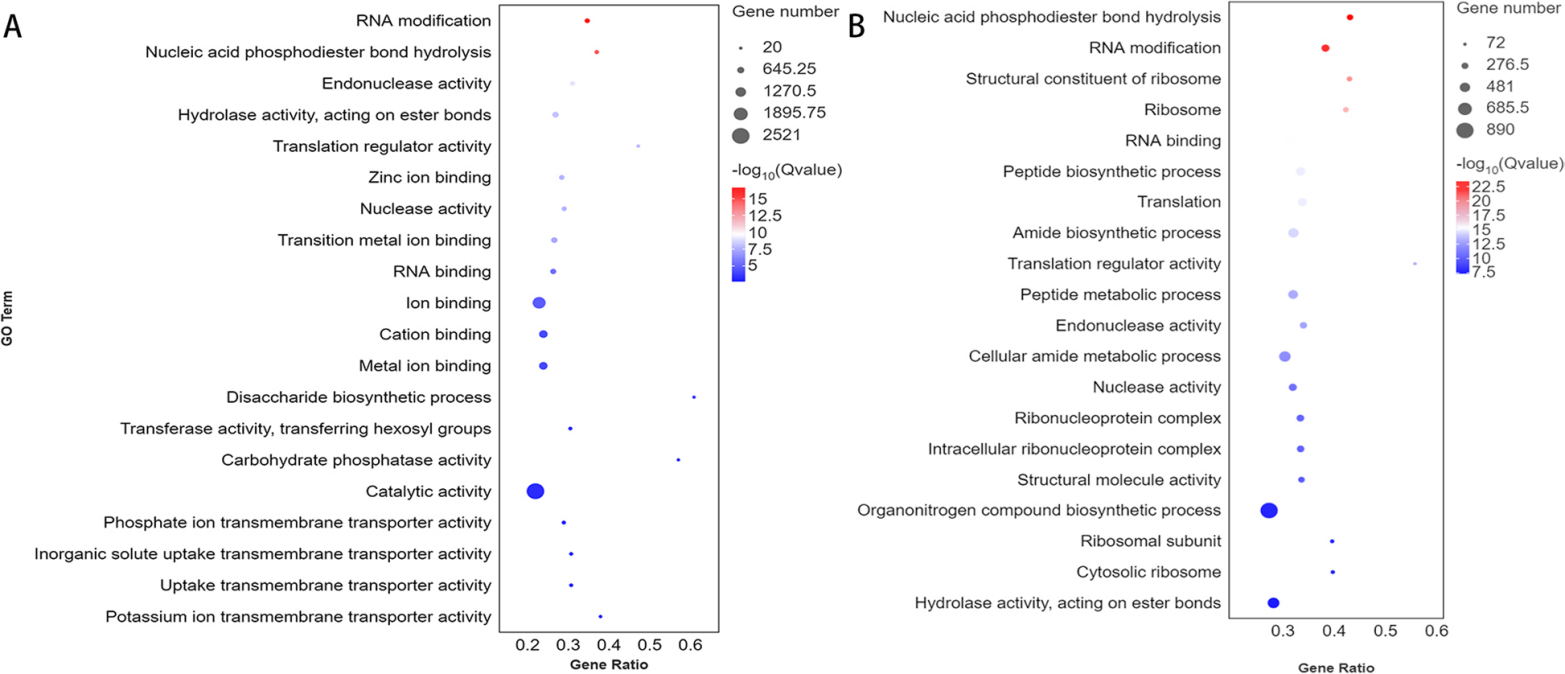
**A**, **B** GO Enrichment Bubble Plot. Bubble size represents the number of differentially expressed genes enriched in the GO term. Bubble color represents the significance of enrichment in the GO term, with larger values indicating greater significance. The x-axis represents the Gene Ratio, which is calculated as the number of differentially expressed genes enriched in the GO term divided by the number of differentially expressed genes enriched in the GO term in the background gene set

Significant enrichment analysis of GO (Gene Ontology) function for differentially expressed genes was performed by comparing the treatment group and control group infected by Xap for 3 days, based on which the top 20 enriched GO entries were selected. The results of the GO enrichment circle maps are shown in Fig. 3A and B. In the differentially expressed genes of resistant varieties infected with Xap for 3 days, GO analysis showed that RNA modification (GO:0009451), nucleic acid phosphodiester bond hydrolysis (GO:0090305), and disaccharide biosynthetic process (GO:0046351) were significantly enriched in the biological process category. In terms of molecular functions, endonuclease activity (GO:0004519), hydrolase activity, acting on ester bonds (GO:0016788), translation regulator activity (GO:0045182), zinc ion binding (GO:0008270), nuclease activity (GO:0004518), transition metal ion binding (GO:0046914), RNA binding (GO:0003723), transferase activity, transferring hexosyl groups (GO:0016758), carbohydrate phosphatase activity (GO:0019203), phosphate ion transmembrane transporter activity (GO:0015114), inorganic solute uptake transmembrane transporter activity (GO:0015318), uptake transmembrane transporter activity (GO:0015563), and potassium ion transmembrane transporter activity (GO:0015079) were all significantly enriched (a total of 17 entries, as shown in Fig. 3A). For susceptible varieties infected with Xap for 3 days, GO enrichment analysis revealed eight entries involved in biological processes, including GO:0005840, among the top 20 enriched GO terms; seven entries involved in molecular functions, including GO:0003735; and five entries related to cell component functions, including GO:0090305 (as shown in Fig. 3B).

Comparing Fig. 3A and B, it can be observed that RNA modification (GO:0009451), nucleic acid phosphodiester bond hydrolysis (GO:0090305), endonuclease activity (GO:0004519), translation regulator activity (GO:0045182), and RNA binding (GO:0003723) were enriched in both resistant and susceptible cultivars. This finding suggests that both resistant and susceptible varieties responded to these biological processes when infected by Xap.

Additionally, some GO terms were exclusively enriched in resistant varieties but not in susceptible varieties. These included catalytic activity (GO:0003824) and hydrolase activity, acting on ester bonds (GO:0016788). These processes may account for the difference between resistant and susceptible varieties in resisting Xap infection.

### KEGG pathway enrichment analysis of differentially expressed genes

**Fig. 4:**
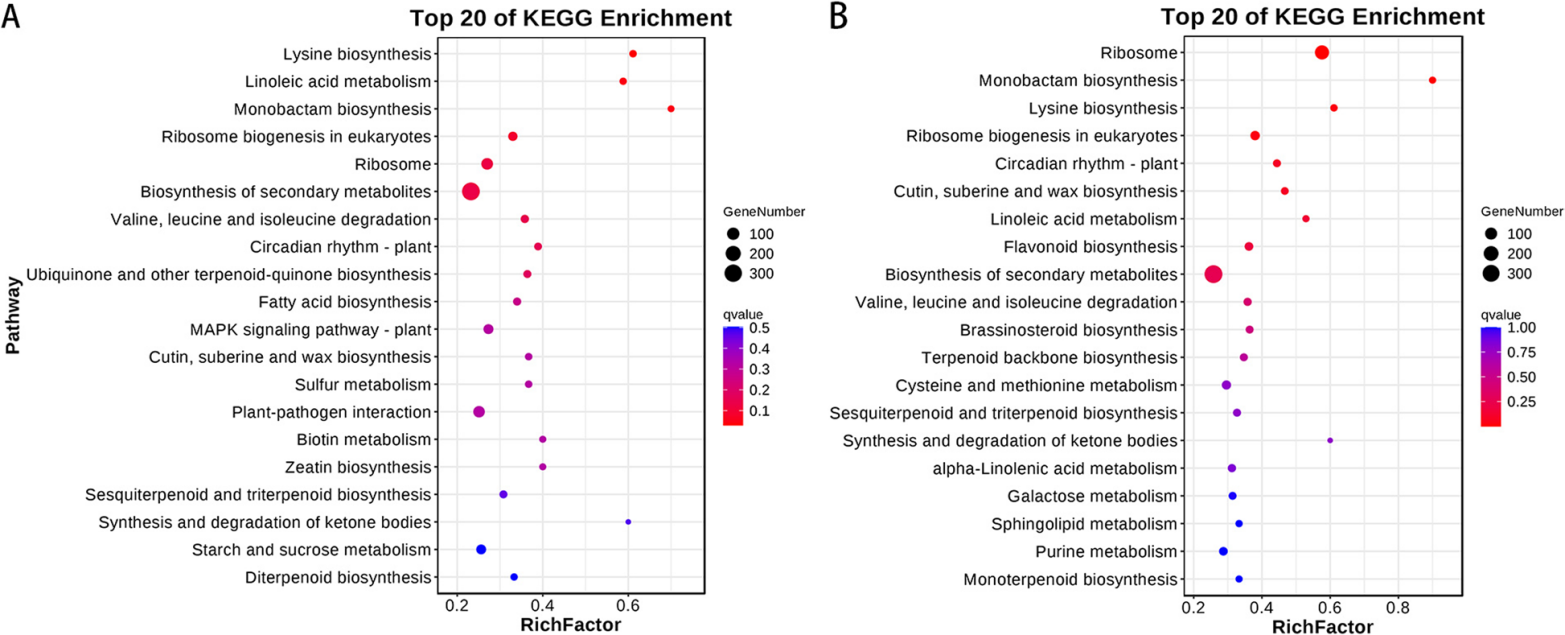
**A**, **B** KEGG Enrichment Bubble Diagram: To plot, use the top 20 pathways with the smallest Q-value. The pathway names are shown on the y-axis, the enrichment factor is shown on the x-axis, and the size of the bubble represents the number of genes enriched in the pathway. The bubbles are colored from red to blue, with smaller Q-values represented by larger bubbles

Figure 4A and B represent the resistant variety R0 vs. R3 control group and susceptible variety S0 vs. S3 control group, respectively. The functions of differentially expressed genes were mainly enriched in the first 20 KEGG pathways.

The differentially expressed genes in resistant and susceptible varieties were enriched in 14 common KEGG pathways, including ribosome, monobactam biosynthesis, lysine biosynthesis, ribosome biogenesis in eukaryotes, circadian rhythm-plant cutin, suberin and wax biosynthesis, linoleic acid metabolism, biosynthesis of secondary metabolites, valine, leucine and isoleucine degradation, sesquiterpenoid and triterpenoid biosynthesis, and synthesis and degradation of ketone bodies. However, the differentially expressed genes in resistant varieties were also enriched in ubiquinone and other terpenoid-quinone biosynthesis, fatty acid biosynthesis, MAPK signaling pathway-plant, sulfur metabolism, plant‒pathogen interaction, biotin metabolism, zeatin biosynthesis, starch and sucrose metabolism, and diterpenoid biosynthesis pathways, which differed from those in susceptible varieties.

### The expression trend of different genes after infection

Analysis of differentially expressed genes (DEGs) revealed 5301 DEGs in R0 vs. R3 and 5549 DEGs in S0 vs. S3. These results indicate that with the increase in infection time for different varieties, the number of genes affected was not significantly different, but the downregulated DEGs were more numerous than the upregulated DEGs.

**Fig. 5:**
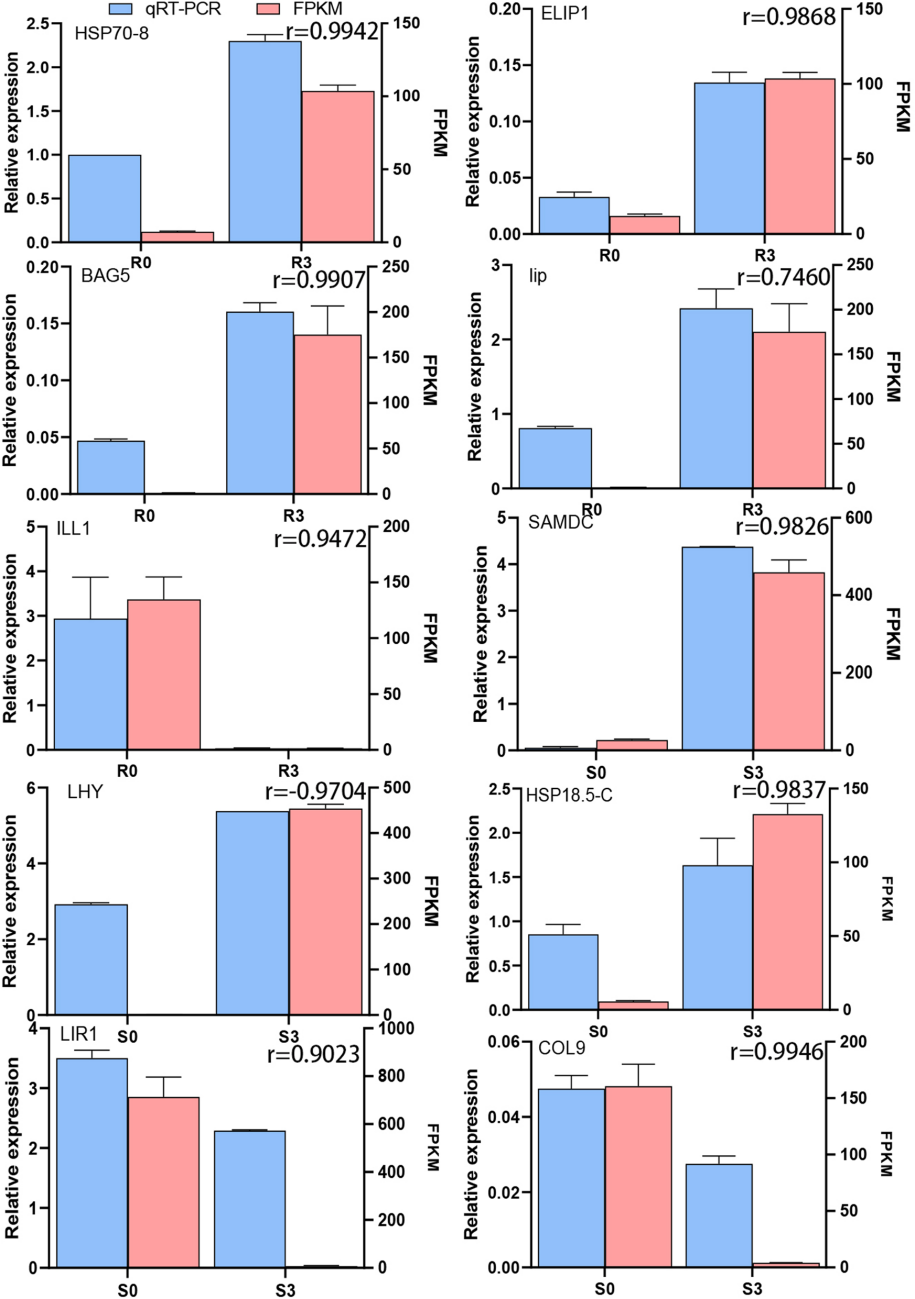
Sequencing verification and qPCR verification. r indicate the correlation coefficient

Trend analysis of DEGs in the two comparison groups showed that five out of 20 gene expression patterns had significant gene expression patterns (*P* < 0.05), including Profile 2, Profile 17, Profile 0, Profile 1, and Profile 7(as shown in Fig. S3). The gene expression of the Profile 2 pattern first decreased and then remained unchanged, while there were 2751 genes in R0 vs. R3 and 3112 genes in S0 vs. S3. The expression of the Profile17 model genes increased first and then remained unchanged, the expression of the Profile 0 model genes decreased gradually, the expression of the Profile 1 model genes decreased first and then increased, and the expression of the Profile 3 model genes first decreased, then remained unchanged, and then decreased again. Most genes were classified into these five significant gene expression patterns. Some genes with different expression patterns were selected for qRT-PCR verification, and the results indicated good consistency with the expression trends observed in the transcriptome data (Fig. 5).

### miRNA sequencing data statistics

To identify resistance-related miRNAs in peach bacterial perforation, a total of 18 miRNA libraries were constructed and subjected to sequencing. In total, 243,250,091 reads were generated. After removing adaptors, low-quality sequences, poly(A), and small RNAs below 18 nt, a set of high-quality small RNA sequences (227,769,021) with a length range of 18–31 nt was obtained (Table S4). These high-quality small RNA sequences were used for subsequent analysis.

Figure S4 displays the size distribution of all small RNAs obtained through sequencing. The length distribution of small RNA reads showed that the length of small RNAs ranged between 18 nt and 35 nt, with approximately 97.14% of small RNAs ranging from 18 nt to 26 nt. Notably, 21 nt (41.26%), followed by 24 nt (23.38%), 22 nt (12.23%), and 20 nt (7.3%), were the most common small RNA sizes.

The sequence distribution displayed in Fig. S4 indicates that most small RNA lengths are primarily 21 nt and 24 nt, and the overall length distribution trend of small RNAs across all samples is consistent. This outcome is similar to that of other plants [56].

### Identification of known and novel miRNAs

To identify conserved/known miRNAs in peach, the filtered reads were compared with miRNAs from other plant species in miRBase. A total of 112 known miRNAs belonging to 70 miRNA families were identified, with varying numbers of members in each family. Among them, 33 miRNA families had only one member, while the miR395 and miR399 families had 14 members each, and the miR156, miR169, miR171, and miR482 families had 9 members each.

Using miRDeep2 software to predict new miRNAs, a total of 111 new miRNAs were obtained. The sequence length of these new miRNAs mainly ranged from 20 nt to 24 nt, with the largest number of new miRNAs having a length of 24 nt (91) and 21 nt having 21 new miRNAs. Of note, novel-m0854-5p and novel-m1186-3p were only expressed in susceptible varieties, while novel-m1276-3p was only expressed in resistant varieties.

### Differential expression analysis of miRNAs

**Fig. 6:**
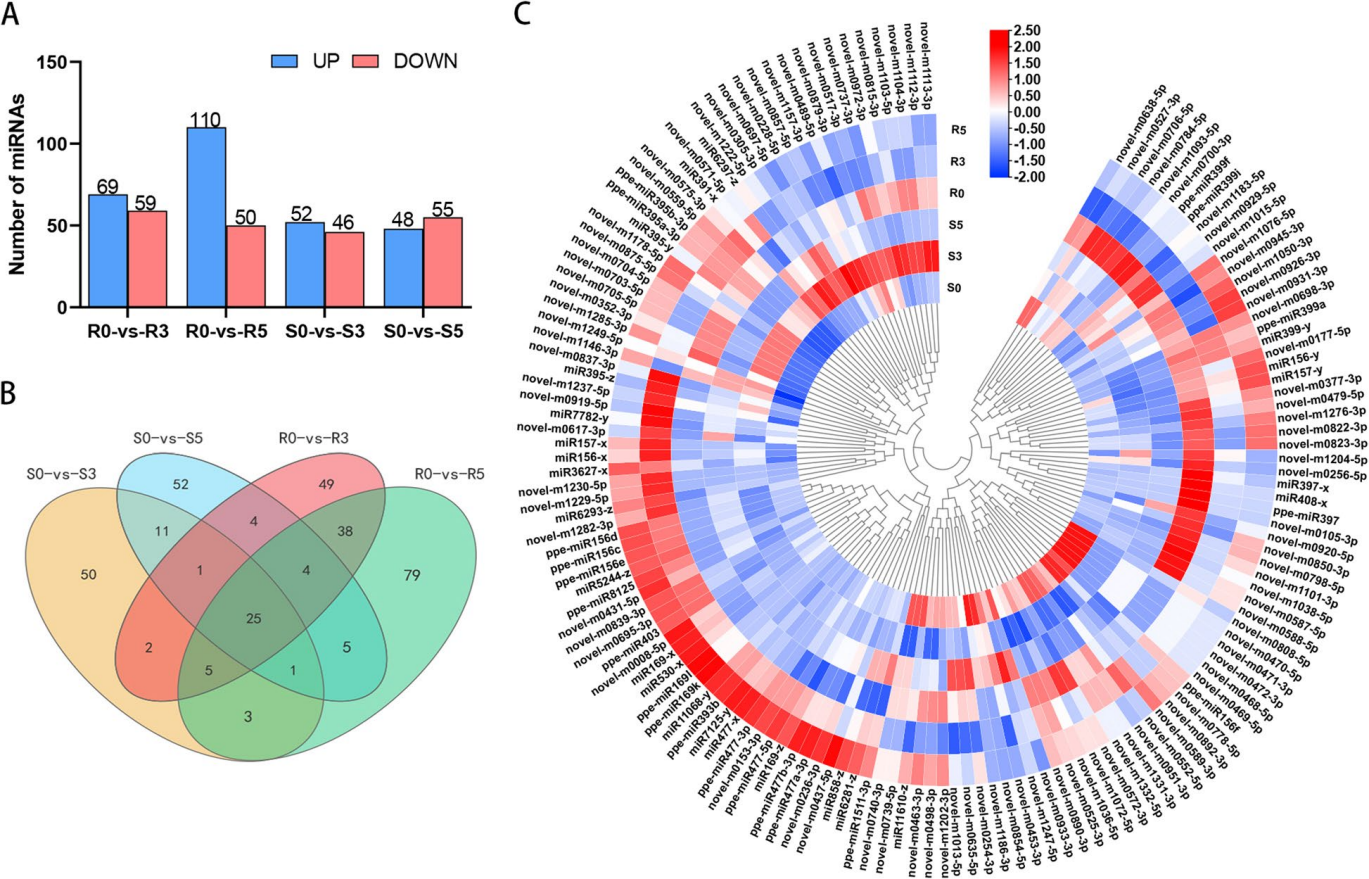
Analysis results of differentially expressed miRNAs. **A** Number of upregulated and downregulated miRNAs in the comparison group. Blue represents upregulated miRNAs, while red represents downregulated miRNAs. **B** Overlapping regions indicate the number of genes shared between groups or comparison groups, while nonoverlapping regions represent genes that are unique to each group or comparison group. **C** Heatmap showing the expression of miRNAs that are significantly and differentially expressed

To identify miRNAs related to bacterial shot hole resistance, differential expression analysis of miRNAs was conducted between the two varieties, and differentially expressed miRNAs were screened. As shown in Figs. 6A and 59 miRNAs (15 known miRNAs and 44 new miRNAs) were differentially downregulated, while 69 miRNAs (54 known miRNAs and 15 new miRNAs) were differentially upregulated in resistant varieties compared to R0 after 3 days of inoculation. Similarly, in susceptible cultivars, 46 miRNAs (15 known miRNAs and 31 new miRNAs) were downregulated, while 52 miRNAs (28 known miRNAs and 24 new miRNAs) were upregulated after 3 days of inoculation compared to S0. The number of differentially expressed genes between each comparison group and the number of common genes between each comparison group were obtained by drawing a Venn diagram of each group of differentially expressed genes, as shown in Fig. 6B. The expression heatmap of differentially expressed miRNAs is shown in Fig. 6C.

### miRNA target gene prediction

As shown in Fig. S5A, there were 1567 miRNAs in R0 corresponding to 4486 target genes and 1524 miRNAs in R3 corresponding to 5010 target genes. Similarly, Fig. S5B shows that there were 1519 miRNAs corresponding to 4398 target genes in S0 and 1515 miRNAs corresponding to 5724 target genes in S3.

### GO enrichment analysis of miRNA target genes

**Fig. 7:**
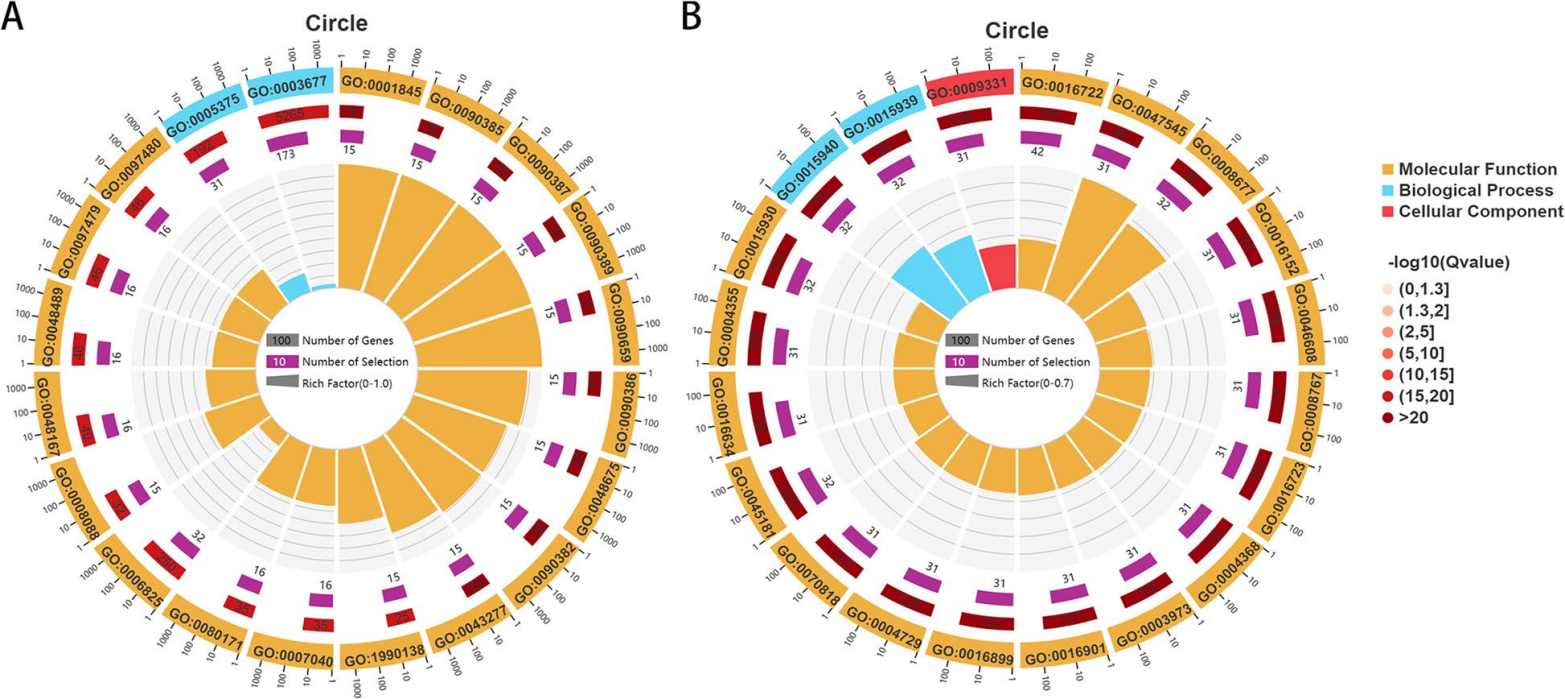
**A**, **B** Enrichment circle plot for differentially expressed miRNA target genes. The first circle shows the top 20 enriched Gene Ontology (GO) terms and the number of genes associated with each term. Different colors represent different GO categories. The second circle displays the number of background genes for each GO term, as well as Q-values. The strip length represents the number of genes, and the color red indicates smaller Q-values. The third circle includes a bar chart displaying the upregulated/downregulated gene ratio, the dark purple bars representing the upregulated gene ratio, and the light purple bars representing the downregulated gene ratio. The values are shown below the chart. The fourth circle shows the Rich Factor values for each GO term

To identify the potential function of Xap infecting miRNAs, the differentially expressed miRNA sequence was submitted to the GO database, and target gene functions were enriched to explore their function during Xap infection. The enrichment circle map of the top 20 functions with the highest enrichment significance (FDR < 0.05) is shown in Fig. 7A. It can be seen that resistant varieties are mainly enriched in biological processes such as phagolysosome assembly (GO:0001845), phagosome-lysosome fusion (GO:0090385), phagolysosome assembly involved in apoptotic cell clearance (GO:0090387), phagosome-lysosome fusion involved in apoptotic cell clearance (GO:0090389), walking behavior (GO:0090659), phagosome maturation involved in apoptotic cell clearance (GO:0090386), and axon extension. Molecular functions mainly include copper ion transmembrane transporter activity (GO:0005375) and DNA binding (GO:0003677).

In Fig. 7B, susceptible varieties were mainly enriched in molecular functions such as oxidoreductase activity, oxidizing metal ions (GO:0016722), 2-hydroxyglutarate dehydrogenase activity (GO:0047545), 2-dehydropantoate 2-reductase activity (GO:0008677), mercury (II) reductase activity (GO:0016152), carotenoid isomerase activity (GO:0046608), UDP-galactopyranose mutase activity (GO:0008767), and oxidoreductase activity, oxidizing metal ions, NAD, or NADP as acceptors (GO:0016723). Biological processes included pantothenate biosynthetic process (GO:0015940) and pantothenate metabolic process (GO:0015939), while glycerol-3-phosphate dehydrogenase complex (GO:0009331) belonged to cellular component.

### miRNA target gene KEGG enrichment analysis

**Fig. 8:**
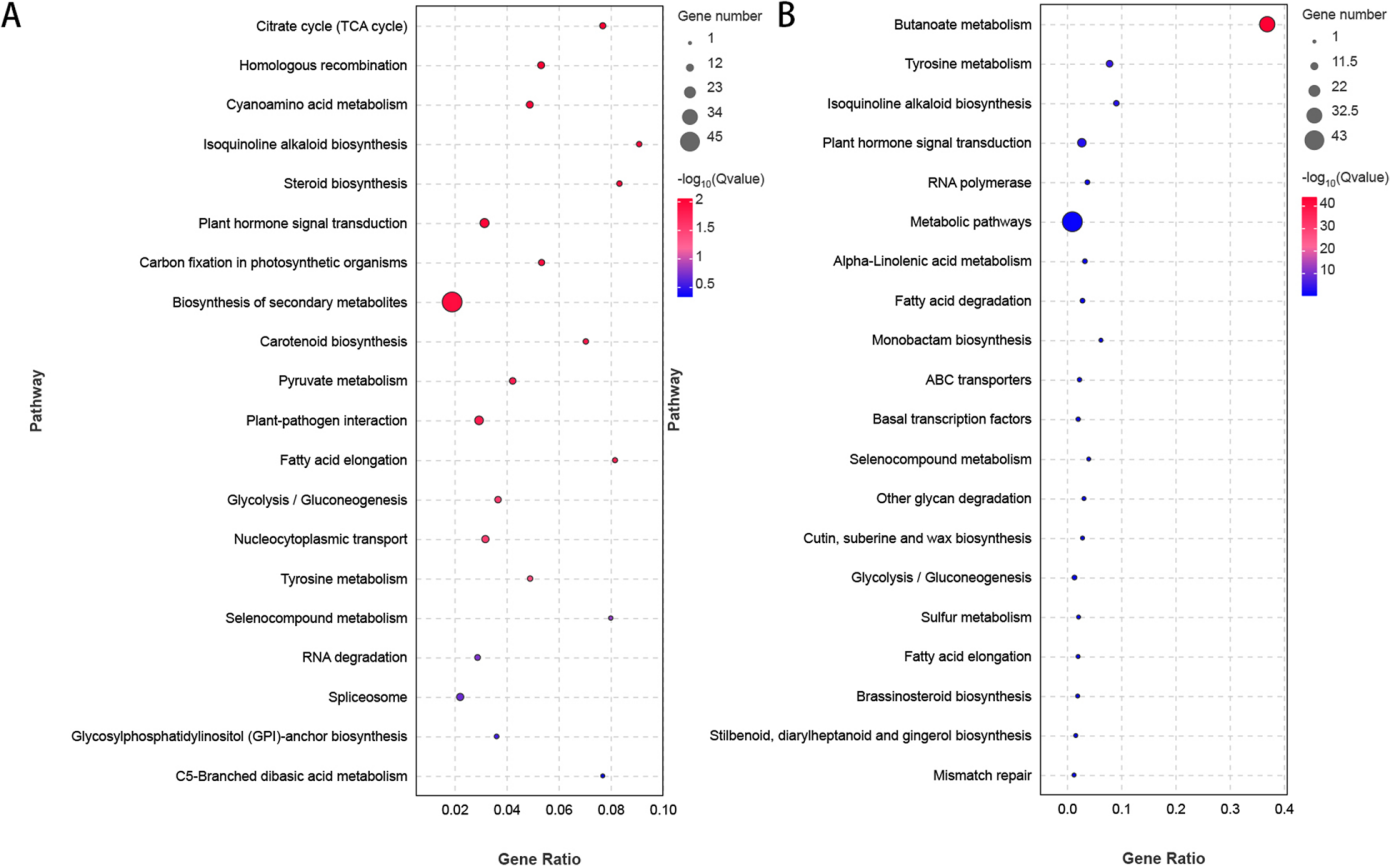
**A**, **B** KEGG enrichment analysis for differentially expressed miRNA target genes. The FDR values indicating the significance of various pathways were plotted, with the x-axis representing gene ratio and the y-axis representing pathway names. Each bubble represents a pathway, with the size of the bubble indicating the number of genes associated with that pathway. The color of the bubble indicates the enrichment significance of the pathway, represented by the size of the FDR value

A total of 99 pathways were obtained through KEGG signal pathway enrichment of target genes, with a total of 61 signaling pathways enriched in the R0 vs. R3 comparison group, as shown in Fig. 8A and B. The top 20 KEGG pathways enriched by the functions of the differential target genes were as follows: 45 genes enriched in the biosynthesis of secondary metabolites (ko01110) pathway and 15 genes enriched in the plant hormone signal transduction (ko04075) pathway. Additionally, there were 14 genes enriched in the plant‒pathogen interaction (ko04626) pathway, while 10 differential genes were enriched in nucleoplasmic transport (ko03013) and spliceosome (ko03040). Furthermore, nine differentially expressed genes were enriched in the homologous recombination (ko03440) and cyanoamino acid metabolism (ko00460) pathways.

Eight DEGs were enriched in the pyruvate metabolism (ko00620) and glycolysis/gluconeogenesis (ko00010) pathways. In the S0 vs. S3 comparison group, a total of 38 signaling pathways were enriched, with the top 20 KEGG pathways enriched by the functions of differential target genes as follows: 43 differential genes enriched in metabolic pathways (ko01100), 31 differential genes enriched in the butanoate metabolism (ko00650) pathway, and 13 differential genes enriched in the plant hormone signal transduction (ko04075) pathway. Additionally, there were 8 differentially expressed genes enriched in the tyrosine metabolism (ko00350) pathway and 5 differentially expressed genes enriched in the isoquinoline alkaloid biosynthesis (ko00950), RNA polymerase (ko03020), alpha-linolenic acid metabolism (ko00592), fatty acid degradation (ko00071), and glycolysis/gluconeogenesis (ko00010) pathways. Furthermore, 3 differentially expressed genes were enriched in the ABC transporter (ko02010) and basal transcription factor (ko03022) pathways.

### qPCR validation

To verify the reliability of the miRNA-seq results, 20 miRNAs were selected from the differentially expressed miRNAs according to the Wayne diagram in Fig. 6B for qPCR-based expression analysis. The results in Fig. S6 show that 5.8 S was used as an internal reference to identify the relative expression of these miRNAs [57]. The expression patterns and overall regulatory trends of most of these 20 miRNAs after Xap treatment were consistent with the high-throughput sequencing data, indicating the reliability of differential expression analysis through high-throughput sequencing (Fig. S6).

### mRNA and miRNA combined analysis

Through joint analysis of miRNAs and mRNAs, more accurate interactions and regulatory mechanisms can be revealed. First, miRNAs can be identified through miRNA sequencing, and their relationship with target genes can be determined. Differential expression analysis of miRNAs and mRNAs can identify key miRNAs and genes. The miRNA‒target gene network regulation map can directly demonstrate the regulation of multiple target genes by a single miRNA, as well as the regulation of a single target gene by multiple miRNAs. Integration of these two types of data enables exploration of regulatory mechanisms involved in gene expression.

### Gene differential expression analysis

**Fig. 9:**
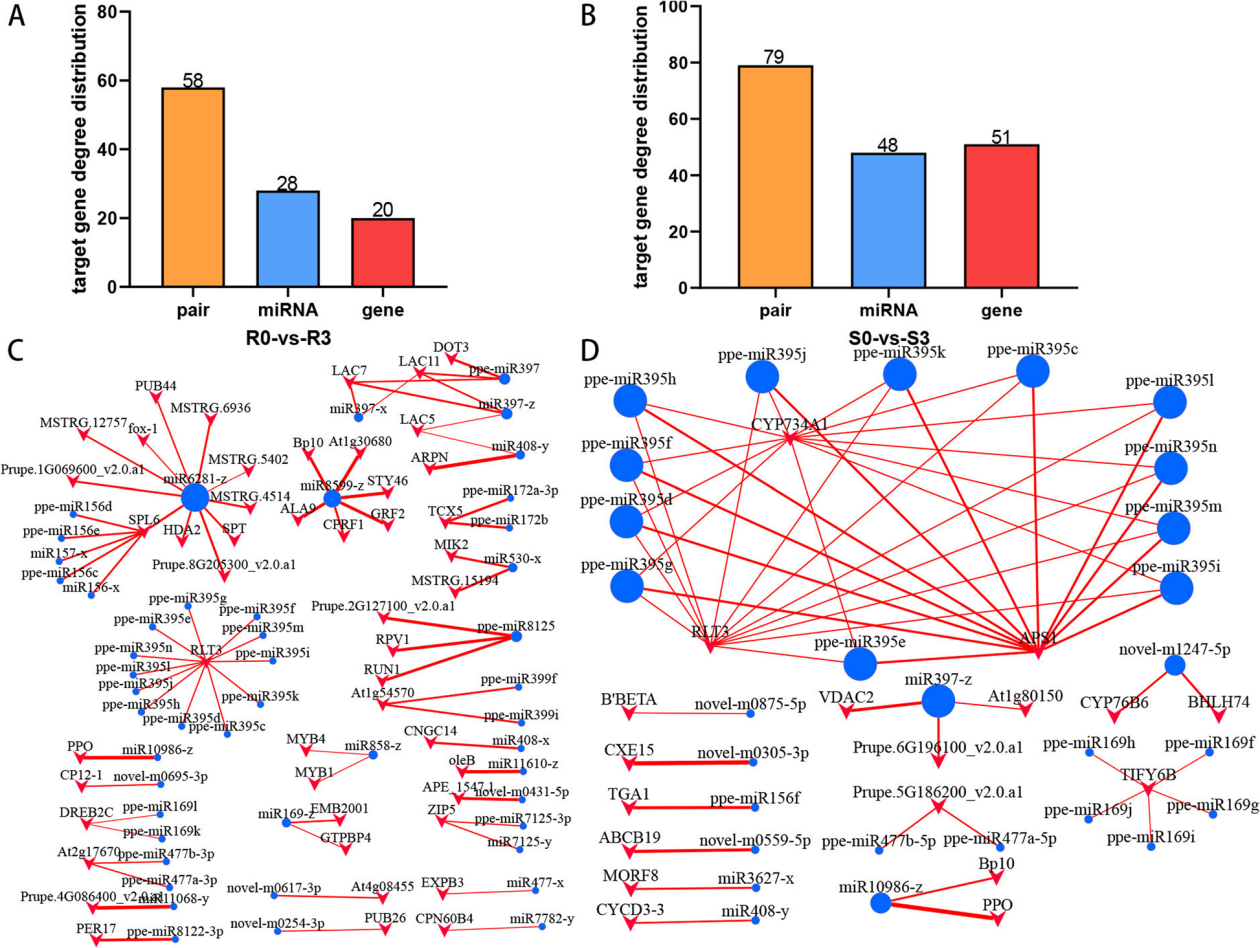
The results of miRNA t arget gene analysis (**A**) and (**B**) predicted the number of miRNAs and target genes of target genes. The abscissa is the name information, pair represents the relationship between miRNA and target gene, miRNA is the predicted target gene miRNA, and target represents the target gene. The ordinate represents the quantity. The abscissa represents the number of bound miRNAs, and the ordinate represents the number of genes regulated by miRNAs. **C** and **D** Negative correlation miRNA‒target gene regulatory network diagram

To reveal the function of miRNAs, potential target genes of miRNAs were predicted, and the resulting target gene information was mapped to previous mRNA sequencing results to obtain information on differentially expressed target genes. Figure 9A and B display 79 relationship pairs in the S0-vs-S3 comparison group, consisting of 48 miRNAs and 51 target genes. Additionally, there were 58 relationship pairs in the R0-vs-R3 comparison group, comprising 28 miRNAs and 20 target genes. Negative correlation miRNA‒target gene pairs were then obtained, and a regulatory network diagram was created using Cytoscape, as shown in Fig. 9C and D.

### Significant enrichment analysis of GO function in the combined analysis

The significant enrichment analysis of GO (Gene Ontology) functions for differentially expressed genes between the treatment group and control group, both inoculated with Xap for 3 days, resulted in 37 selected GO terms, as shown in Fig. S7A and B. In the differentially expressed genes of the resistant variety after 3 days of inoculation, 19 enriched GO terms were identified under biological processes, including localization (GO:0051179), rhythmic process (GO:0048511), metabolic process (GO:0008152), biological regulation (GO:0065007), locomotion (GO:0040011), cellular process (GO:0009987), cellular component organization or biogenesis (GO:0071840), and single-organism process (GO:0044699). For molecular function, seven enriched GO terms were observed, mainly transporter activity (GO:0005215), nucleic acid binding transcription factor activity (GO:0001071), binding (GO:0005488), catalytic activity (GO:0003824), antioxidant activity (GO:0016209), electron carrier activity (GO:0009055), and transcription factor activity, protein binding (GO:0000988). The enriched GO terms for cell components included extracellular region part (GO:0044421), extracellular matrix (GO:0031012), extracellular region (GO:0005576), macromolecular complex (GO:0032991), cell part (GO:0044464), cell (GO:0005623), and organelle (GO:0043226), among the total of eleven GO terms.

In contrast, the results of GO enrichment analysis for differentially expressed genes of the susceptible variety after 3 days of inoculation (Fig. S5B) showed the selection of 30 GO terms, including 15 involved in biological processes (GO:0008152, etc.), six in molecular function (GO:0000988, etc.), and nine involved in cell component-related functions (GO:0044421, etc.).

Comparing Fig. S7. A and B, it can be seen that some GO terms, such as metabolic process (GO:0008152) under biological processes, nucleic acid binding transcription factor activity (GO:0001071) under molecular function, and membrane part (GO:0044425) under cell component, were enriched in both resistant and susceptible varieties after Xap inoculation, indicating that both varieties respond to these biological processes when infected by Xap.

### KEGG pathway enrichment analysis of differentially expressed genes for combined analysis

Figure S8A and B present the top 20 KEGG pathways that are predominantly enriched in the functions of differentially expressed genes in the resistant variety R0 vs. R3 comparison group and susceptible variety S0 vs. S3 comparison group, respectively. Both resistant and susceptible varieties share some common pathways that are enriched by differential genes, such as biosynthesis of secondary metabolites (ko01110), metabolic pathways (ko01100), isoquinoline alkaloid biosynthesis (ko00950) and tyrosine metabolism. In contrast to susceptible varieties, the differentially expressed genes in resistant varieties were also enriched in ribosome biogenesis in eukaryotes (ko03008), RNA degradation (ko03018), nucleocytoplasmic transport (ko03013), phenylpropanoid biosynthesis (ko00940), and plant‒pathogen interaction (ko04626). In contrast, the differentially expressed genes in susceptible varieties were enriched in additional pathways, including plant hormone signal transduction (ko04075), monocobactam biosynthesis (ko00261), selenocompound metabolism (ko00450), sulfur metabolism (ko00920), ABC transporters (ko02010), brassinosteroid biosynthesis (ko00905), purine metabolism (ko00230), and mRNA surveillance pathway (ko03015).

## Discussion

Compared to research on other fruits, research on the peach transcriptome and miRNAs involved in disease resistance is relatively limited. This study employs transcriptome data and small RNA association analysis to explore target genes associated with miRNAs that may play a role in peach resistance to diseases. By performing principal component analysis (PCA) on the transcriptome data, we observed clear separation between different varieties and treatments, indicating that these factors significantly influence gene expression patterns (Fig. S1A). This aligns well with the observed differences in phenotype features (Fig. 1A). These findings suggest that gene expression patterns may play a crucial role in specific varieties and treatments and may form the basis for the formation of different phenotype features. This PCA analysis provides important clues for a deeper understanding of the relationship between gene expression and phenotype. In a previous study, transcriptome analysis of *X. arboricola* pv *pruni*-infected peach leaves revealed that 263 genes exhibited significant differential expression [58]. Gervasi et al. conducted RNA-Seq analysis on two distinct peach varieties and observed that differentially expressed genes were principally enriched in signal transduction and secondary metabolism [59]. This study identified 5301 differentially expressed genes in the R0 vs. R3 comparison group and 5549 differentially expressed genes in the S0 vs. S3 comparison group. Enrichment analysis revealed that differentially expressed genes in resistant varieties were mainly associated with pathways such as fatty acid biosynthesis, MAPK signaling pathway-plant, sulfur metabolism, plant‒pathogen interaction, biotin metabolism and zeatin biosynthesis. These findings suggest that peach may enhance resistance by regulating genes involved in signaling pathways after Xap infection. When banana, eggplant, turkey berry, and sunflower plants are infected by pathogens, many genes are involved in the plant‒pathogen interaction, with signal transduction and resistance-related metabolic pathways being the main processes [60–62]. These research results are consistent with the disease resistance mechanisms discussed in this article.

Transcriptomic analysis has proven to be a valuable tool for identifying differentially expressed genes associated with plant defense responses. However, recent evidence has suggested that small noncoding RNAs, such as miRNAs, also play crucial roles in regulating immune responses against pathogen invasion. In plants, miRNAs have been found to be involved in various biological processes, such as development, stress response, and disease resistance. This study analyzed miRNA responses to Xap infection in peach leaves, with three biological replicates set up for each treatment to ensure data credibility. High-throughput sequencing analysis revealed that peach leaves contain a large number of miRNAs, and 448 miRNAs were identified, with 112 known miRNAs and 111 novel miRNAs detected. Among the reported miRNAs, the most highly expressed families are miR395 and miR399 [63, 64]. Among the 111 novel miRNAs identified, novel-m0854-5p and novel-m1186-3p were found only in susceptible varieties, while novel-m1276-3p was exclusively expressed in resistant varieties. These novel miRNAs might be induced by Xap infection. To predict target genes of differentially expressed miRNAs, online prediction software was employed. A total of 6728 target genes, mainly involved in metabolic processes, single-organism processes, binding, and cellular processes, were obtained through statistical analysis for both the R0 vs. R3 and S0 vs. S3 comparison groups. Other studies have identified miRNAs that are specifically induced upon pathogen infection and may play important roles in plant defense. For instance, miR160 and miR164 were found to be induced upon fungal infections [65]. Plants protect themselves by recognizing microbial infections and initiating corresponding immune responses. Among them, endogenous small RNAs are considered to be important regulators, which are involved in the immune response of plants against pathogens [66]. Campo et al. reported a new rice miRNA, osa-miR7695, which negatively regulates an alternatively spliced transcript of OsNramp6 [67]. Zhang et al. showed that expressing a target mimic of miR156fhl-3p led to enhanced rice blast disease resistance without a yield penalty [26]. Qin et al. identified pathogen-responsive miRNAs in apple leaves challenged by Alternaria alternata apple pathotype (AAAP) [68].

Combined analyses of transcriptomic data and small RNA sequencing have enabled researchers to investigate gene regulation and signaling networks underlying the plant defense response mediated by both mRNAs and miRNAs. The integration of these two high-throughput technologies facilitates the elucidation of regulatory mechanisms and target identification, thus holding great promise for improving crop resistance against pathogens. The results obtained from the combined analysis of transcriptome and miRNA data showed 79 relationship pairs in the S0-vs-S3 comparison group, consisting of 48 miRNAs and 51 target genes. In the R0-vs-R3 comparison group, there were 58 relationship pairs, including 28 miRNAs and 20 target genes. Moreover, the study found that a single miRNA can regulate multiple target genes, and a single target gene can also be regulated by multiple miRNAs, such as SPL6, RLT3, APS1, CYP734A1, and other genes investigated in this study. Among these targeted genes, some are related to plant growth and development, while others are pathogenesis-related genes that are specifically expressed under pathogen stress. Additionally, the expression of the miR156 family in this study was upregulated, while the expression of the target gene SPL6 was downregulated. These results are consistent with research demonstrating that miR156v may contribute to plant disease resistance by downregulating SPL6 expression [69]. This study investigated the response mechanism of peach to Xap infection, which is under stringent regulation by different metabolic pathways. Transcriptome and miRNA combined analysis revealed that TIFY6B might be involved in signal transduction, and the expression level of miR169 was downregulated, whereas the miR395 family was upregulated in both susceptible and resistant varieties. The expression of target genes was downregulated accordingly. These findings suggest that the response mechanism of peach to Xap infection involves not only intricate plant self-regulation but also multiple metabolic pathways. Enrichment analysis indicated that biosynthesis of secondary metabolites (ko01110), metabolic pathways (ko01100), isoquinoline alkaloid biosynthesis (ko00950), and tyrosine metabolism were the four most enriched KEGG pathways among differentially expressed genes in both susceptible and resistant varieties. In contrast to susceptible varieties, differentially expressed genes in resistant varieties were also enriched in ribosome biogenesis in eukaryotes (ko03008) and other related pathways. Conversely, differential genes in susceptible varieties were enriched in plant hormone signal transduction (ko04075), among others. The study identified several genes that may be involved in disease resistance, as shown in Fig. S9. These include *TIFY6B*, *Prupe.4G041800_v2.0.a1 (PPO)*, *Prupe.4G041500_v2.0.a1 (PPO)*, *APS1*, *CNGC14 CYP734A1*, *CPN60B4*, *GTPBP4*, *B’BETA*, *ABCB19*, *Prupe.4G041700_v2.0.a1 (PPO)*, *Prupe.4G041900_v2.0.a1 (PPO), Prupe.4G086400_v2.0.a1, PER17*, and *CYCD3-3*. Among these genes, PPO has been previously reported in studies investigating disease resistance [70, 71].

This study focused on two different peach varieties. Expanding the sample dataset to investigate disease resistance mechanisms in more peach varieties can provide useful references for breeding programs. This paper only analyzed the interaction between miRNA and mRNA in a specific biological process, but the regulatory network between miRNA and mRNA is quite complex. In the future, the regulatory network mechanism under various conditions can be explored further.

## Conclusion

The aim of this study was to compare the mRNA and miRNA of two peach tree cultivars, ‘Yanbao’ (resistant) and ‘Yingzui’ (susceptible), after inoculation with Xap to identify miRNAs and target genes associated with peach tree resistance. By combining the mRNA and miRNA data, several pairs of relationships between miRNAs and target genes were identified, providing insights into the regulation of gene expression in response to Xap infection. Through literature reports and enrichment analysis, several target genes related to resistance were identified, including *SPL6, TIFY6B*, and *Prupe.4G041800_v2.0.a1* (*PPO*).Overall, this study identified several candidate genes and miRNAs associated with peach tree resistance against peach bacterial spot disease. These findings improve our understanding of the resistance mechanism in peach trees and provide valuable information for future studies on disease resistance in fruit trees.

### Supplementary Information

**Supplementary Material 1.**

**Supplementary Material 2.**

## Data Availability

These data can be found here: National Center for Biotechnology Information (NCBI) BioProject database under accession number PRJNA989552 (https://www.ncbi.nlm.nih.gov/sra/?term=PRJNA989552) and PRJNA1017963 (https://www.ncbi.nlm.nih.gov/sra/?term=PRJNA1017963).
