# Supplementary material for: Combined analysis of mRNA and miRNA transcriptomes reveals the regulatory mechanism of Xanthomonas arboricola pv pruni resistance in Prunus persica
Source: BMC Genomics. 2024 Feb 27;25:214. doi: 10.1186/s12864-024-10113-8 (PMC10898114; doi:10.1186/s12864-024-10113-8)
Supplement: Supplementary file 1 — Supplementary Material 1. [file 12864_2024_10113_MOESM1_ESM.docx]

**Supplementary Information**

**Identification methods and grading standards of resistance of varieties**

In order to measure the area of disease lesions, leaf samples are collected 5 days after inoculation. After the lesions fully fall off and form disease holes, a transparent grid film ruler is used to measure the area of the disease holes. The transparent grid film ruler has a size of 100 mm × 100 mm, with a minimum scale of 1 mm. Each grid area is 1 mm^2^. By covering the disease hole with the transparent grid film ruler, the number of grids occupied by the disease hole is counted. If the area is larger than 0.5 mm^2^, it is counted as 1 grid. If the area is smaller than 0.5 mm^2^, it is counted as 0 grids.

The disease severity level is defined based on the size of the disease hole area as follows:

Level 0: Disease hole area between 0 and 1 mm^2^

Level 1: Disease hole area between 1.1 and 2 mm^2^

Level 2: Disease hole area between 2.1 and 3 mm^2^

Level 3: Disease hole area between 3.1 and 4 mm^2^

Level 4: Disease hole area between 4.1 and 5 mm^2^

Level 5: Disease hole area larger than or equal to 5.1 mm2

Resistance evaluation; Input the disease severity levels into the disease index calculation formula to obtain the disease index:

$$\text{Infection index}=\frac{\sum\text{（Number of disease holes at all levels * the grade of the disease）}}{\text{The total number of holes investigated * the highest grade of disease}}\times100$$

According to the infection index, the resistance levels are classified as follows:

High Resistance (HR): Infection Index < 15;

Resistance (R): 15 ≤ Infection Index < 20;

Moderate Resistance (MR): 20 ≤ Infection Index < 30;

Susceptible (S): 30 ≤ Infection Index < 50;

High Susceptibility (HS): Infection Index ≥ 50;

| Number | Name | Infection Index | Level |
| --- | --- | --- | --- |
| 1 | Lixia Hong | 2.5 | HR |
| 2 | Lian Huang | 8.33 | HR |
| 3 | Xinjiang Huang Rou Tao | 8.33 | HR |
| 4 | Yan Bao | 8.48 | HR |
| 5 | Du Bai Feng | 9.17 | HR |
| 6 | Hong Shou | 10 | HR |
| 7 | Xuancheng Tian Tao | 16.67 | R |
| 8 | Li Ge Lan Te | 17.14 | R |
| 9 | Zhong You 13 Hao | 17.5 | R |
| 10 | Crimsonbaby | 17.58 | R |
| 11 | Yu Lu Pan Tao | 20 | R |
| 12 | Tian Li Guang | 20 | R |
| 18 | JST2 | 30 | MR |
| 19 | Zhubo 5 Hao | 30 | MR |
| 20 | Xiao Hong Hua | 35.83 | S |
| 21 | Huan Xiang | 37.5 | S |
| 22 | Zao Hong | 43.33 | S |
| 23 | Shuang Fu | 45 | S |
| 24 | Xia Ji Tao | 80 | HS |
| 25 | Fenghua Pan Tao | 80 | HS |
| 26 | NJT-50 | 91.52 | HS |
| 27 | yingzui | 95.76 | HS |
| 28 | Guang He Tao | 97.5 | HS |

**Fig. S1**(**A**) , (**B**)PCA analysis and correlation heat map

**Fig. S2** (**A**), (**B**), (**C**) and (**D**) Comparison of differences between different comparison groups. The volcano map shows that the abscissa represents the logarithm of the difference between the two groups, the ordinate represents the negative Log10 value of the FDR of the two groups, and the red expression is upregulated.) and blue (downregulated expression) points indicate that the expression of genes is different (the criterion is FDR < 0.05, and the difference is more than twice), and the black point is no difference.

**Fig. S3** (**A**), (**B**) Analysis of gene expression trends in different comparison groups. Sorted in descending order by the number of genes expressed.

**Fig. S4** The type and proportion of sRNA in each sample. The abscissa represents the tag length (nt); the ordinate represents tag abundance; different colors represent different types of sRNA.

**Fig. S5** Statistics of predicted target genes for miRNAs.

**Fig. S6** Sequencing verification and qPCR verification. (**A**) and (**B**) MiRNA expression changes caused by Xap infection.

**Fig. S7** (**A**), (**B**) Target gene GO enrichment results. The ordinate of the graph is the GO term, and the abscissa is the number of genes enriched in the GO term. The longer the column is, the greater the number of enriched target genes.

**Fig. S8** GO (**A**), (**B**) target gene KEGG enrichment results. FDR values of different pathway significance were plotted. The abscissa is the RichFactor, and the ordinate is the Pathway. Each bubble represents a pathway. The size of the bubble represents the number of genes contained in the pathway. The color of the bubble represents the enrichment significance of the pathway, that is, the size of the FDR value.

**Fig. S9** Heatmap of Differentially Expressed Target Genes.

**
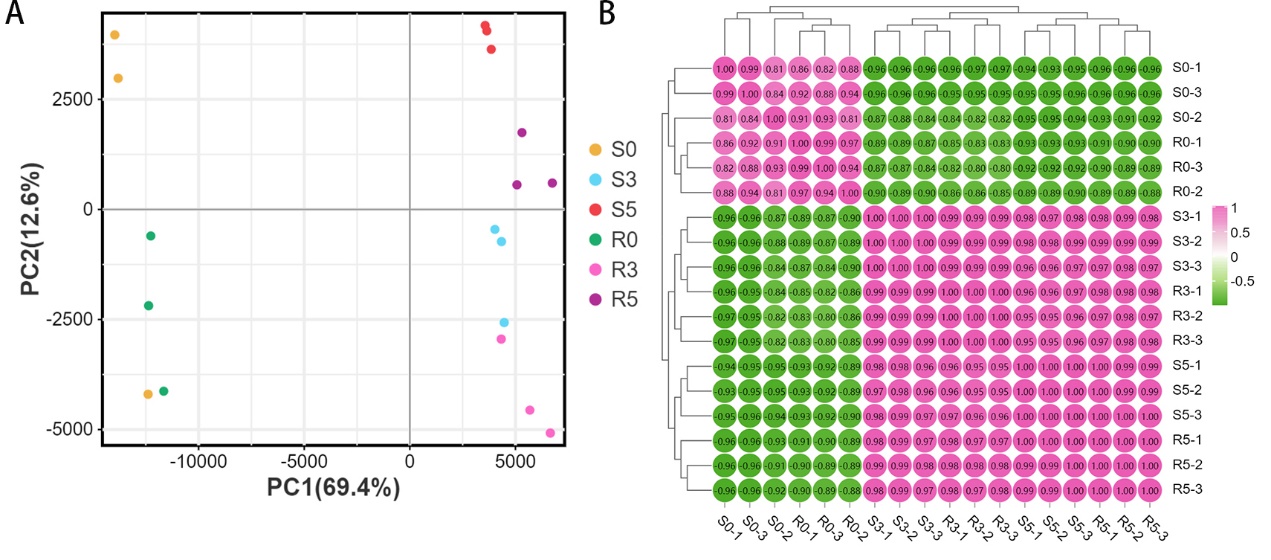
**

**Fig. 1S**


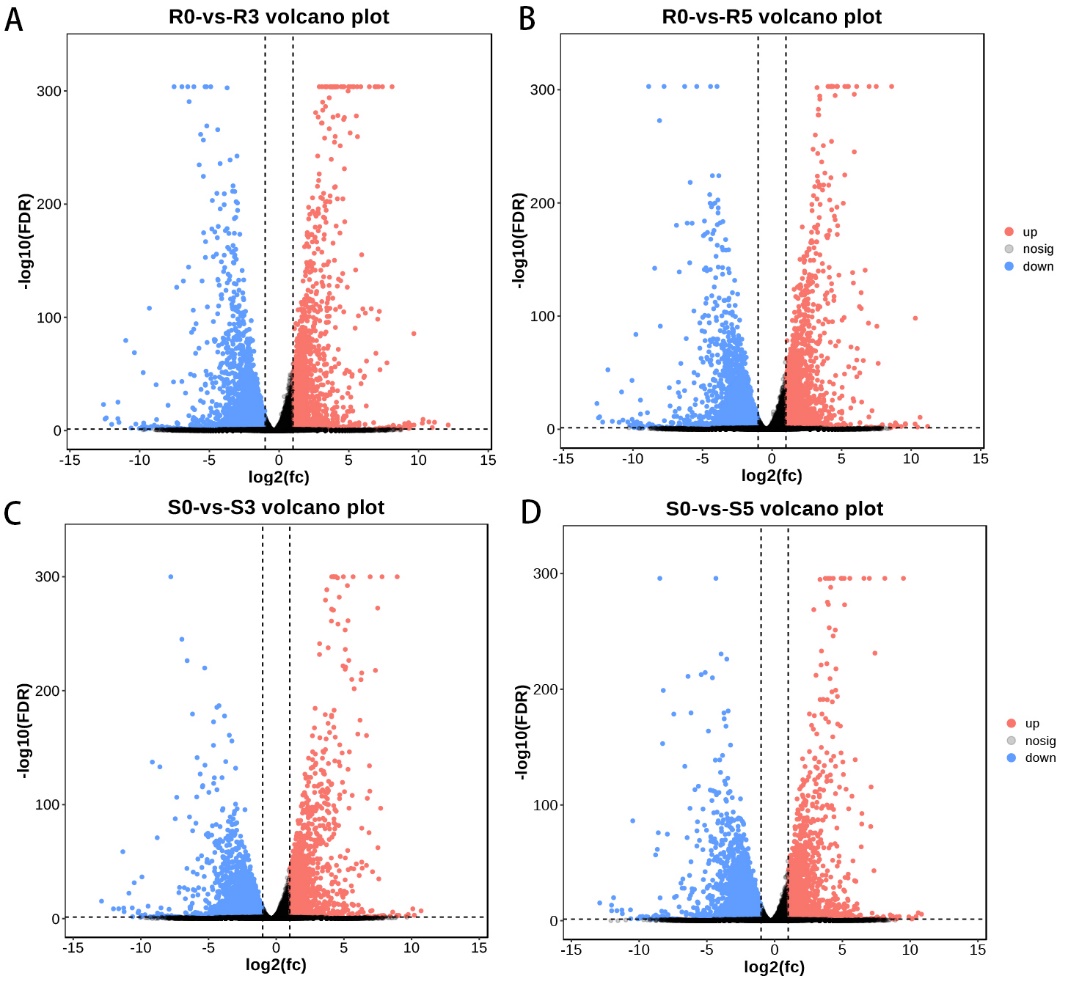


**Fig. S2**


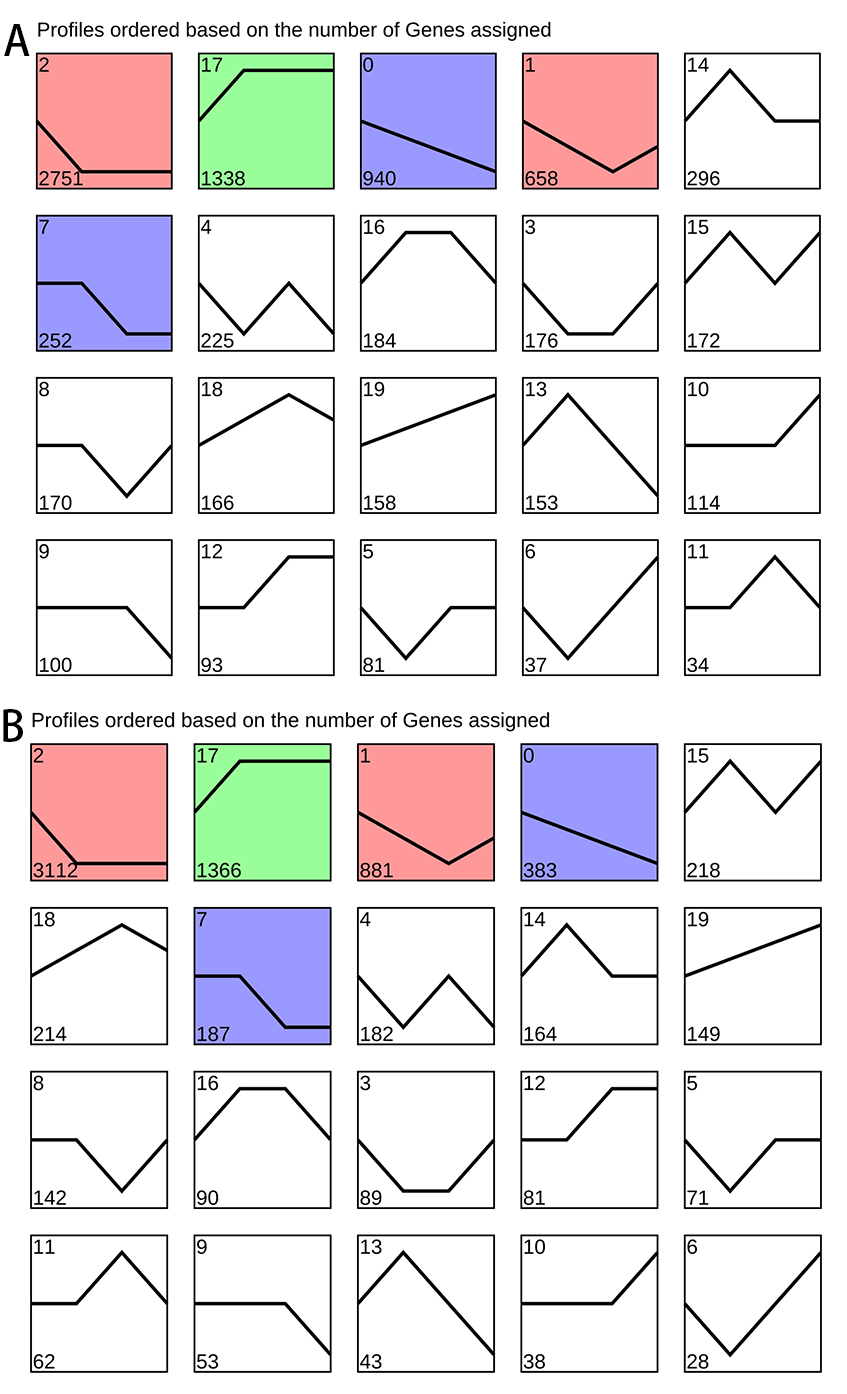


**Fig. S3**


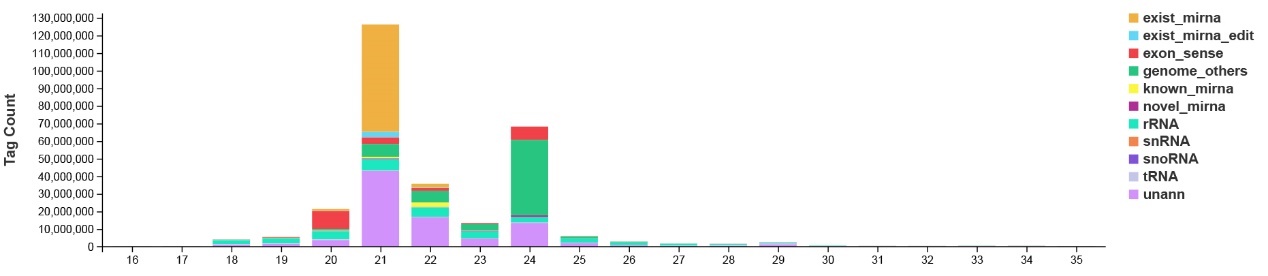


**Fig. S4**


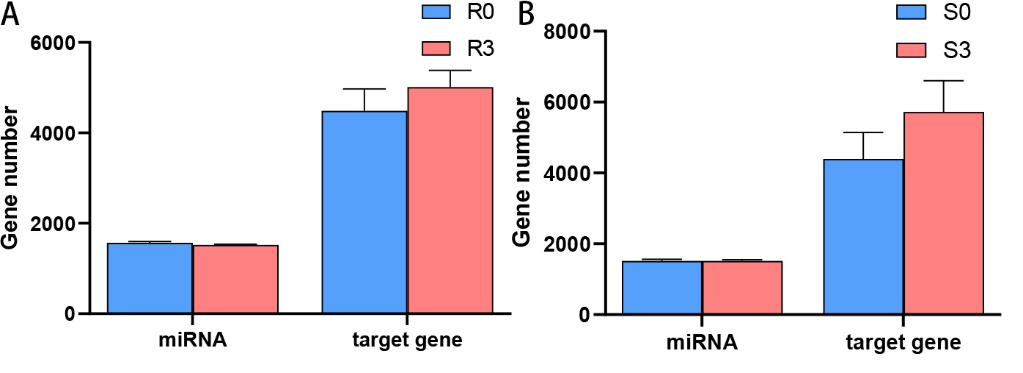


**Fig. S5**


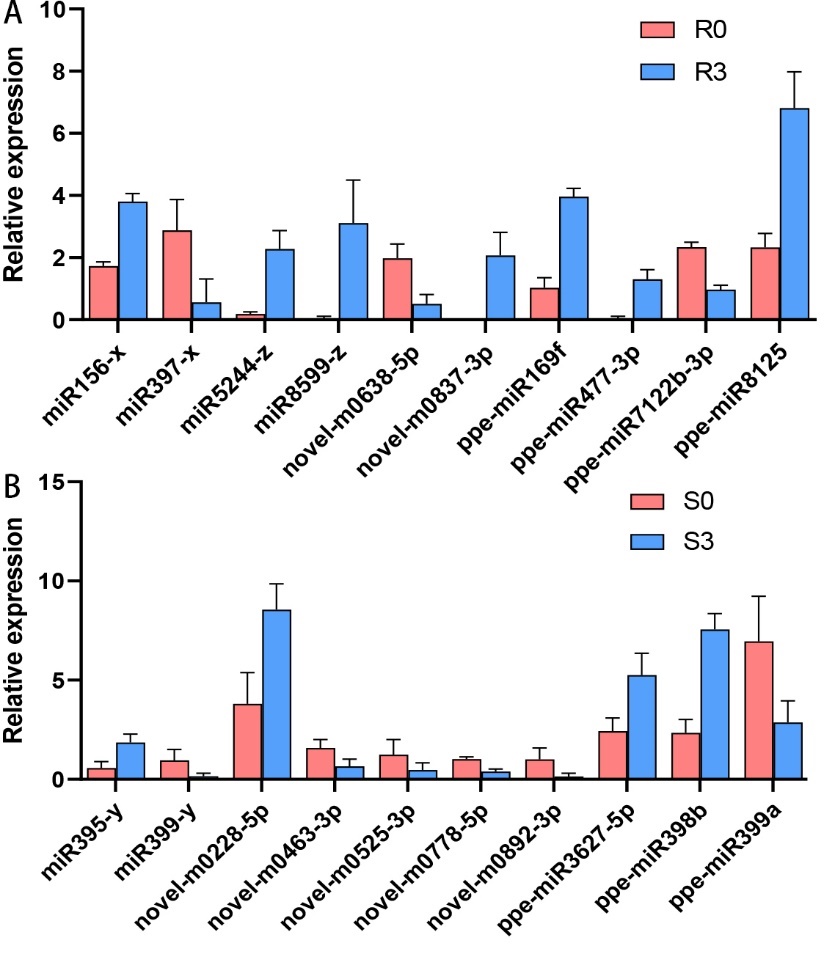


**Fig. S6**


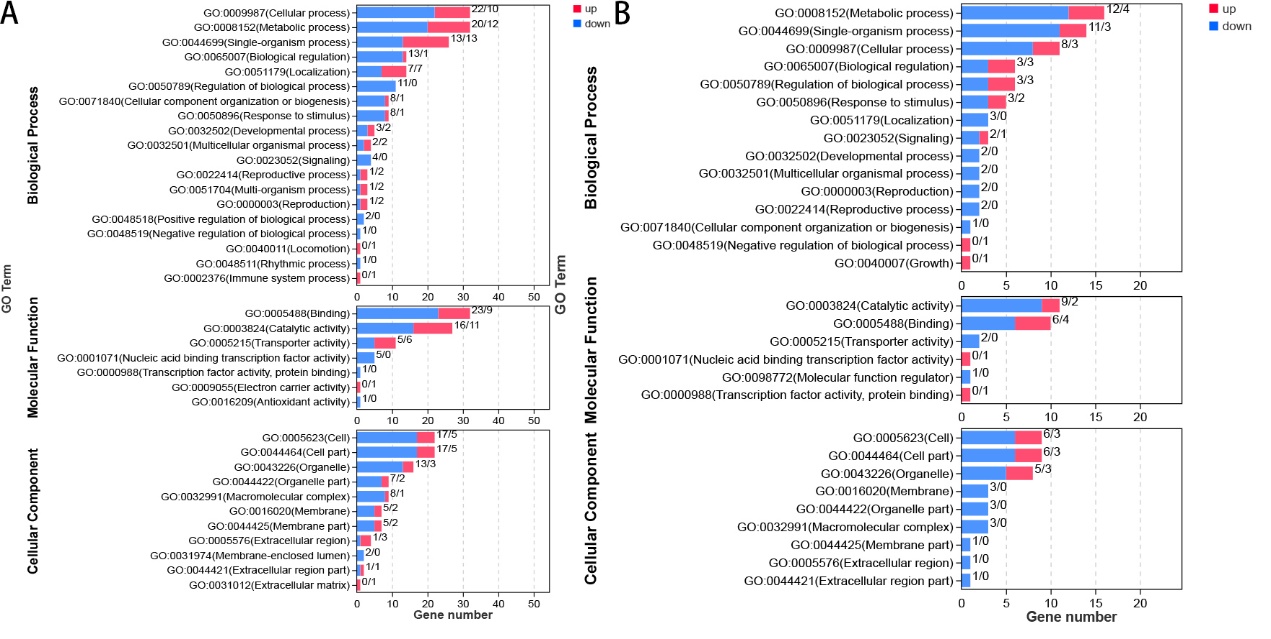


**Fig. S7**


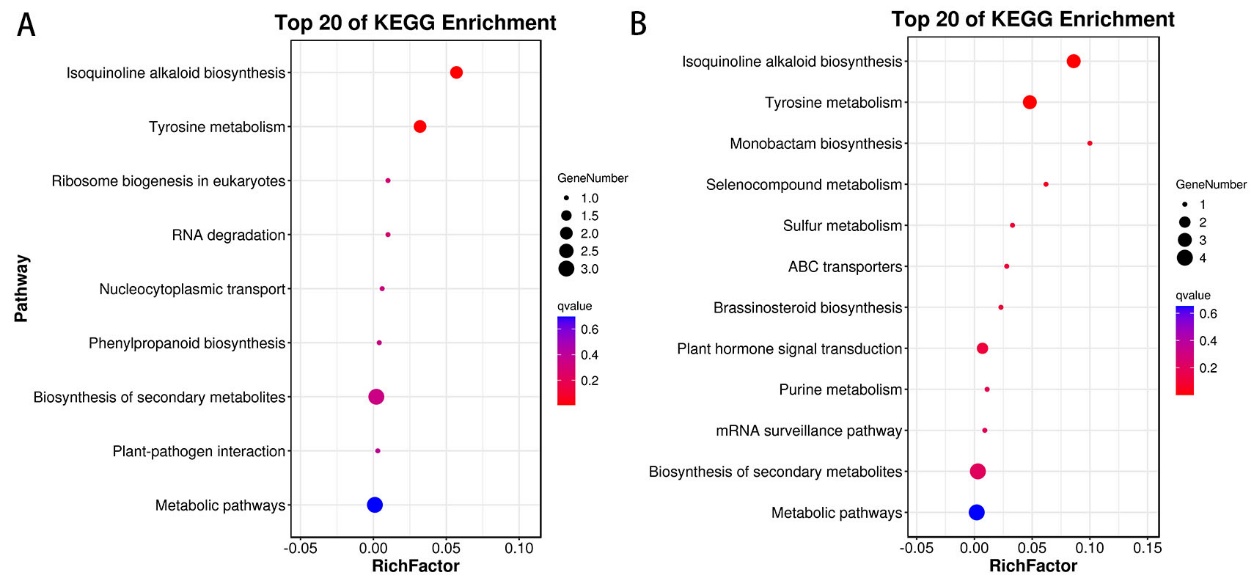


**Fig. S8**


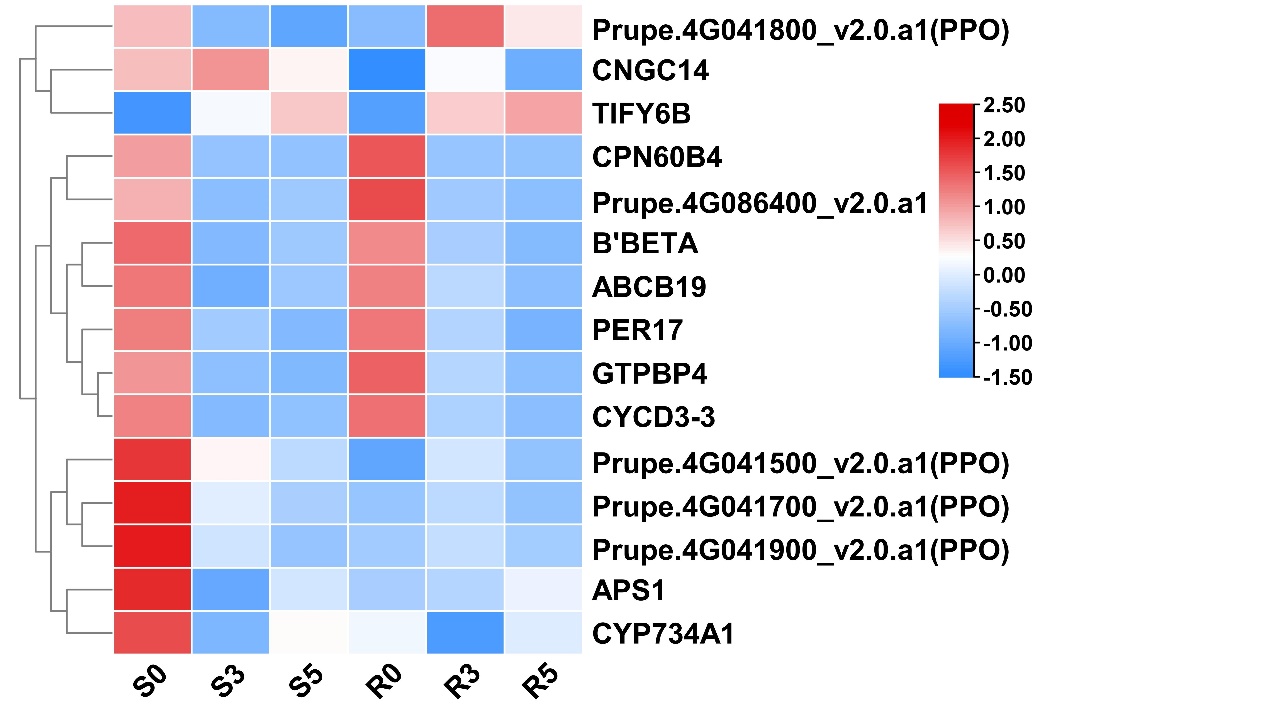


**Fig. S9**

**Table S1 List of primers used in this study.**

| **Gene** |  | Primer sequence(5'-3') |
| --- | --- | --- |
| HSP70-8 | Forward primer | TCAAAAATCTGTGTGTCTCGGC |
|  | Reverse primer | CACGACCCCTTTGCATTTCC |
| ELIP1 | Forward primer | ACCCGTCTGTATAGTGAGCA |
|  | Reverse primer | GTAAACGGAACATCTTACAGACAA |
| BAG5 | Forward primer | ACCCGTCTGTATAGTGAGCA |
|  | Reverse primer | GTAAACGGAACATCTTACAGACAA |
| lip | Forward primer | TGAAACCCCAAAACCCCCATAA |
|  | Reverse primer | AAGGGTTCTCGTGCCTGATT |
| ILL1 | Forward primer | ACTCGTATGCAGATGCAATGT |
|  | Reverse primer | AGAACCGTAGCCTTTCGAGC |
| SAMDC | Forward primer | CAGTCATACCCTCATCGT |
|  | Reverse primer | AGCCTTCTCCCTGTCCAA |
| LHY | Forward primer | GCCTTTCAAGCACTATTC |
|  | Reverse primer | CATCATTGTCTCCTACGG |
| HSP18.5-C | Forward primer | ACCCATTCACAGATTTCC |
|  | Reverse primer | AAAGAAGACCTCACCACC |
| LIR1 | Forward primer | GAGTACGACAGCCCCAAGAC |
|  | Reverse primer | AGACCCTGCAGCAATCATCAA |
| COL9 | Forward primer | GGCGTGCTTATGTTTATC |
|  | Reverse primer | TTGCCTCTTATGTGCTGA |
| actin | Forward primer | CAGATCATGTTTGAGACCTTCAATGT |
|  | Reverse primer | CATCACCAGAGTCCAGCACAAT |

**Table S2** Sequencing data filtering statistics table

| Sample | RawDatas | CleanData(%) | LowQuality(%) | polyA(%) | N(%) |
| --- | --- | --- | --- | --- | --- |
| S0-1 | 45717476 | 45546098 (99.63%) | 157154 (0.34%) | 0 (0.00%) | 0 (0.00%) |
| S0-2 | 51625232 | 51406360 (99.58%) | 201268 (0.39%) | 0 (0.00%) | 0 (0.00%) |
| S0-3 | 45046534 | 44828556 (99.52%) | 199886 (0.44%) | 0 (0.00%) | 34 (0.00%) |
| S3-1 | 40963778 | 40815708 (99.64%) | 134232 (0.33%) | 0 (0.00%) | 0 (0.00%) |
| S3-2 | 48871212 | 48686576 (99.62%) | 169634 (0.35%) | 0 (0.00%) | 0 (0.00%) |
| S3-3 | 42547628 | 42395614 (99.64%) | 138104 (0.32%) | 0 (0.00%) | 0 (0.00%) |
| S5-1 | 49207548 | 49021676 (99.62%) | 170006 (0.35%) | 0 (0.00%) | 0 (0.00%) |
| S5-2 | 36035540 | 35902468 (99.63%) | 118414 (0.33%) | 0 (0.00%) | 12 (0.00%) |
| S5-3 | 43429140 | 43275454 (99.65%) | 136958 (0.32%) | 0 (0.00%) | 0 (0.00%) |
| R0-1 | 40358372 | 40195222 (99.60%) | 148874 (0.37%) | 0 (0.00%) | 0 (0.00%) |
| R0-2 | 42599130 | 42396214 (99.52%) | 184244 (0.43%) | 0 (0.00%) | 38 (0.00%) |
| R0-3 | 44350784 | 44139408 (99.52%) | 192522 (0.43%) | 0 (0.00%) | 36 (0.00%) |
| R3-1 | 50623078 | 50437944 (99.63%) | 164502 (0.32%) | 0 (0.00%) | 0 (0.00%) |
| R3-2 | 47689608 | 47519236 (99.64%) | 152018 (0.32%) | 0 (0.00%) | 0 (0.00%) |
| R3-3 | 47374114 | 47215538 (99.67%) | 141892 (0.30%) | 0 (0.00%) | 0 (0.00%) |
| R5-1 | 47790412 | 47607640 (99.62%) | 163822 (0.34%) | 0 (0.00%) | 2 (0.00%) |
| R5-2 | 49010132 | 48833948 (99.64%) | 158774 (0.32%) | 0 (0.00%) | 8 (0.00%) |
| R5-3 | 46889768 | 46720322 (99.64%) | 152992 (0.33%) | 0 (0.00%) | 0 (0.00%) |

**Table S3** Statistical analysis of sequence alignment results between sequencing data and selected reference genome

| Sample | Total | Unmapped(%) | Unique_Mapped(%) | Multiple_Mapped(%) | Total_Mapped(%) |
| --- | --- | --- | --- | --- | --- |
| S0-1 | 45430420 | 1384154 (3.05%) | 42486965 (93.52%) | 1559301 (3.43%) | 44046266 (96.95%) |
| S0-2 | 51297866 | 1646208 (3.21%) | 47762519 (93.11%) | 1889139 (3.68%) | 49651658 (96.79%) |
| S0-3 | 44702672 | 1709058 (3.82%) | 41685594 (93.25%) | 1308020 (2.93%) | 42993614 (96.18%) |
| S3-1 | 40705670 | 1190817 (2.93%) | 38180741 (93.80%) | 1334112 (3.28%) | 39514853 (97.07%) |
| S3-2 | 48576178 | 1433933 (2.95%) | 45543966 (93.76%) | 1598279 (3.29%) | 47142245 (97.05%) |
| S3-3 | 42277098 | 1172277 (2.77%) | 39675103 (93.85%) | 1429718 (3.38%) | 41104821 (97.23%) |
| S5-1 | 48850916 | 1476770 (3.02%) | 45772953 (93.70%) | 1601193 (3.28%) | 47374146 (96.98%) |
| S5-2 | 35774300 | 1173845 (3.28%) | 33484177 (93.60%) | 1116278 (3.12%) | 34600455 (96.72%) |
| S5-3 | 42851534 | 1309634 (3.06%) | 40140063 (93.67%) | 1401837 (3.27%) | 41541900 (96.94%) |
| R0-1 | 40096288 | 1281347 (3.20%) | 37656983 (93.92%) | 1157958 (2.89%) | 38814941 (96.80%) |
| R0-2 | 42279952 | 1399260 (3.31%) | 39747944 (94.01%) | 1132748 (2.68%) | 40880692 (96.69%) |
| R0-3 | 44018480 | 1258859 (2.86%) | 41476049 (94.22%) | 1283572 (2.92%) | 42759621 (97.14%) |
| R3-1 | 50259608 | 1451151 (2.89%) | 47134254 (93.78%) | 1674203 (3.33%) | 48808457 (97.11%) |
| R3-2 | 47356674 | 1404075 (2.96%) | 44404977 (93.77%) | 1547622 (3.27%) | 45952599 (97.04%) |
| R3-3 | 47068364 | 1401185 (2.98%) | 44141718 (93.78%) | 1525461 (3.24%) | 45667179 (97.02%) |
| R5-1 | 47449438 | 1563243 (3.29%) | 44393320 (93.56%) | 1492875 (3.15%) | 45886195 (96.71%) |
| R5-2 | 48664666 | 1463959 (3.01%) | 45649065 (93.80%) | 1551642 (3.19%) | 47200707 (96.99%) |
| R5-3 | 46544048 | 1313651 (2.82%) | 43767364 (94.03%) | 1463033 (3.14%) | 45230397 (97.18%) |

**Table S4** Statistics of each sample data filtering and removing joints

| id | clean_reads | high_quality | 3'adapter  _null | insert_null | 5'adapter  contaminants | polyA | clean_tags |
| --- | --- | --- | --- | --- | --- | --- | --- |
| S0-1 | 14857144 | 14717512 | 45151 | 265174 | 7359 | 420 | 13972066 |
| S0-2 | 10021754 | 9961272 | 15949 | 125596 | 4327 | 285 | 9535709 |
| S0-3 | 14375377 | 14236602 | 16668 | 400872 | 11061 | 613 | 13141942 |
| S3-1 | 13496944 | 13354332 | 22137 | 188176 | 7187 | 648 | 12757174 |
| S3-2 | 12718026 | 12592272 | 33730 | 208047 | 7582 | 737 | 11925169 |
| S3-3 | 15131087 | 14990323 | 22272 | 249398 | 7853 | 726 | 14267425 |
| S5-1 | 14401293 | 14256086 | 142000 | 212622 | 14423 | 628 | 13322701 |
| S5-2 | 14767304 | 14621607 | 49602 | 287077 | 9497 | 566 | 13657043 |
| S5-3 | 13513993 | 13389167 | 16356 | 263720 | 12109 | 590 | 12276573 |
| R0-1 | 15036015 | 14878505 | 33719 | 178391 | 7005 | 466 | 14315875 |
| R0-2 | 11327062 | 11268814 | 20016 | 85507 | 4020 | 260 | 10924643 |
| R0-3 | 14910418 | 14769249 | 22807 | 126295 | 5968 | 433 | 14312247 |
| R3-1 | 14328225 | 14028373 | 234450 | 127682 | 8318 | 625 | 13263150 |
| R3-2 | 11421924 | 11359090 | 89865 | 85680 | 5072 | 594 | 10855760 |
| R3-3 | 10268861 | 10211830 | 26796 | 90595 | 4791 | 338 | 9870427 |
| R5-1 | 15812966 | 15649969 | 30175 | 154372 | 11920 | 419 | 14693879 |
| R5-2 | 13863057 | 13736876 | 22198 | 192710 | 8973 | 616 | 12891813 |
| R5-3 | 12998641 | 12884622 | 19104 | 196424 | 11570 | 612 | 11785425 |
|  | 243250091 | 240906501 | 862995 | 3438338 | 149035 | 9576 | 227769021 |
